# Evaluation of protected benzoic acid on growth performance, nutrient digestibility, and gut health indices in starter pigs

**DOI:** 10.1093/tas/txad111

**Published:** 2023-09-14

**Authors:** Alex Outlaw, Alexandra Gachman, Haejin Kim, Xiangyi Xu, Zhigang Tan, Zhonghua Qin, Xianfeng Peng, Marko Rudar

**Affiliations:** Department of Animal Sciences, Auburn University, Auburn, AL 36849, USA; Department of Animal Sciences, Auburn University, Auburn, AL 36849, USA; Department of Animal Sciences, Auburn University, Auburn, AL 36849, USA; Department of Animal Sciences, Auburn University, Auburn, AL 36849, USA; Department of Animal Sciences, Auburn University, Auburn, AL 36849, USA; Department of Animal Sciences, Auburn University, Auburn, AL 36849, USA; Guangzhou Insighter Biotechnology Co., Ltd., Guangzhou, Guangdong 510664, China; Department of Animal Sciences, Auburn University, Auburn, AL 36849, USA

**Keywords:** benzoic acid, cytokine, digestibility, intestine, pig, weaning

## Abstract

Benzoic acid is a common alternative for antibiotic and zinc oxide use in nursery diets. Free benzoic acid (**BZA**) is often supplied, but this form is absorbed before it can exert any effect on distal segments of the gut. The study aimed to evaluate the effects of protected benzoic acid on growth performance, nutrient digestibility, plasma metabolites, and gut health indices in starter pigs. A total of 192 pigs were weaned at 28 ± 1 d age (initial body weight, 8.72 ± 1.13 kg). Pens were assigned to one of four treatment diets (*n* = 8 pens per treatment): (1) no additive (**NC**), (2) free benzoic acid (BZA; 0.6%), (3) protected benzoic acid (**BC50**; 0.2%, supplied at a ratio of one to three equivalents of BZA), and (4) antibiotic growth promoter (**AGP**; Carbadox, 50 ppm). Diets were fed for three weeks over two periods (period 1, 7 d; period 2, 14 d). Body weight and feed intake were measured for each period. Feces were collected at the end of each period to determine apparent total tract digestibility (**ATTD**) of organic matter (**OM**), gross energy (**GE**), and crude protein (**CP**). One pig per pen was euthanized per period to determine plasma metabolites; jejunum and ileum morphology; jejunum, ileum, and colon cytokine abundance; and jejunum, ileum, and colon tight junction protein expression. The AGP group had increased average daily gain (**ADG**) and average daily feed intake (**ADFI**) compared to other groups in period 1 and overall (*P* < 0.05); however, ADG and ADFI of the BC50 group was intermediate between the NC and BZA groups and the AGP group in period 2. The ATTD of OM, GE, and CP were greater in the AGP group compared to the NC and BC50 groups (*P* < 0.05), whereas the BZA group was intermediate. Jejunum and ileum villus height and crypt depth increased from period 1 to period 2 (*P* < 0.01) but were similar across groups. Ileum and colon tumor necrosis factor-α (**TNF-α**) abundances were greater, whereas colon interleukin (**IL**)-1β and colon and ileum IL-8 abundances were less, in the AGP group compared to the BZA group (*P* < 0.05); the NC and BC50 groups exhibited intermediate TNF-α, IL-1β, and IL-8 abundance in the ileum and colon. Jejunum cytokine abundance did not vary among groups but declined from period 1 to period 2 (*P* < 0.05). Tight junction protein expression also did not vary among groups. In summary, protected BZA supported a slight increase in growth performance in starter pigs, suggesting its potential as an alternative feed additive in nursery diets.

## INTRODUCTION

The first month of postnatal life is a critical period for intestinal development in pigs (Moeser et al., 2017). However, current industry practice of weaning pigs at about 21 days of age leads to marked structural and functional changes in the small intestine. Weaning alters intestinal architecture, modifies the microbiome, and impairs nutrient utilization, mucosal immunity, and gut barrier capacity (Pluske et al., 2018). Collectively, these changes limit growth performance and increase the incidence of postweaning diarrhea, a multifactorial disease and significant cause of morbidity and mortality in the nursery (Rhouma et al., 2017; Eriksen et al., 2021). Despite improvements in management strategies for starter pigs, the postweaning growth check continues to hinder pork producers and is a major source of lost revenue to the swine industry.

The inclusion of antibiotic growth promoters and pharmacological amounts of zinc oxide in nursery diets are both highly effective strategies to mitigate weaning stress. Concerns about the indiscriminate use of antimicrobials in animal production systems and links to the emergence of antimicrobial-resistant pathogens (Tang et al., 2017) have paved the way for restrictions and bans on the use of medically important antimicrobials for animal growth promotion across the United States, Canada, and European Union (Casewell et al., 2003; FDA, 2012; PHAC, 2015). More recently, the European Union has phased out zinc oxide due to concerns about environmental zinc accumulation (Jensen et al., 2016) and development of antimicrobial resistance (Slifierz et al., 2015; Vahjen et al., 2015). Eliminating both antimicrobials and zinc oxide as tools to help manage the weaning transition underlines the need for alternative strategies to improve pig performance and reduce the incidence of enteric disease. Organic acids are widely used as alternatives to antibiotic growth promoters (**AGP**) and zinc oxide (Lopez-Galvez et al., 2021). Organic acids are thought to promote pig performance by increasing gastric acidification and protein digestion. Consequently, reduced flow of undigested protein to the hindgut limits protein fermentation to ammonia, branched-chain fatty acids, and biogenic amines that adversely affect gut health (Nyachoti et al., 2006; Gilbert et al., 2018). Organic acids capable of diffusing across bacterial membranes may also have direct antimicrobial activity (Warnecke and Gill, 2005). Among organic acids, benzoic acid is reported to increase growth performance, nutrient digestibility, and gut health indices (Kluge et al., 2006; Torrallardona et al., 2007; Diao et al., 2016; Kiarie et al., 2018) and shows robust in vitro antimicrobial activity against *Escherichia coli* and *Salmonella* (Knarreborg et al., 2002; Friedman et al., 2003). Benzoic acid is excreted in urine as its glycine conjugate, hippuric acid, in pigs, leading to acidification of urine and a reduction in ammonia emissions from manure (Murphy et al., 2011).

Free organic acids, including benzoic acid, are absorbed rapidly in the stomach and proximal small intestine (Kristensen et al., 2009). Organic acids protected in a carbohydrate, lipid, or protein matrix that prevent their immediate absorption can exert their effects in the distal small intestine and hindgut. Protected organic acid blends, excluding benzoic acid, are reported to increase growth performance and nutrient digestibility in starter, growing, and finishing pigs (Upadhaya et al., 2016, 2018; Nguyen et al., 2019). Average daily gain and feed efficiency are marginally increased in pigs supplied a protected organic acid blend, including benzoic acid, compared to pharmacological zinc oxide (Correa et al., 2021). However, there is limited information available on the effect of protected benzoic acid alone. By understanding the potential benefits of protected benzoic acid, we can develop effective nutritional strategies to improve pig performance and reduce the reliance on antimicrobials and zinc oxide. The objective of this study was to determine the effects of protected benzoic acid on growth performance, nutrient digestibility, plasma metabolites, and gut health indices in starter pigs. We hypothesized that protected benzoic acid will enhance gut health in nursery pigs by modulating intestinal morphometry, short-chain fatty acid (**SCFA**) concentrations, proinflammatory cytokine abundance, and tight junction protein expression.

## MATERIALS AND METHODS

### Animals and Housing

All animal procedures were approved by the Institutional Animal Care and Use Committee at Auburn University (PRN 2022-4057). Starter pigs (Yorkshire × Duroc × Hampshire cross) were obtained from the Swine Research and Education Center at Auburn University. The animal trial was conducted in three replicate blocks (June to July 2022, August to September 2022, and January to February 2023). A total of 192 pigs with initial body weight 8.72 ± 1.13 kg (mean ± standard deviation) over three blocks were weaned at 28 ± 1 d age and assigned to mixed-sex pens (6 pigs per pen; 32 total pens). Pigs were assigned to pens according to body weight (**BW**) and sex (i.e., equal mix of barrows and gilts per pen). All pigs were housed in the same nursery room of 18 pens across blocks. Pens were separated by powder-coated vertical steel rods and equipped with a single-sided five-hole nursery feeder (76 cm length × 29 cm width; AP-SN*305; Automated Production Systems, Sterling Heights, MI) and cup waterer (270650; QC Supply, Schuyler, NE). Each pen provided 3 m^2^ area (1.5 m × 2 m; 0.5 m^2^ area per pig) on snap-joint polypropylene flooring. Pens were elevated above 36-cm deep pits that are filled with recycled water and flushed weekly for waste management. Initial room temperature was set to 29 °C and was reduced by 1.7 °C per week for 3 weeks; room temperature was maintained by automated ventilation fans. Pigs were vaccinated against acute rhinitis, pneumonia, and erysipelas at 7 d and 28 d age (Rhini Shield TX4; Elanco Animal Health, Greenfield, IN). The source swine herd was negative for coccidiosis and other enteric diseases.

### Animal Diets

Eight treatment diets were formulated over two periods (period 1, 7 d; period 2, 14 d) to meet or exceed estimated requirements for all nutrients for 10 to 25 kg pigs (NRC, 2012). Two basal diets, corresponding to period 1 and period 2, were formulated based on corn and soybean meal. Four treatment diets were formulated per period (period 1, Table 1; period 2, Table 2) from the corresponding basal diet with no additive (**NC**: negative control), free benzoic acid (**BZA**; 0.60%), protected benzoic acid (Benzocal-50, **BC50**; 0.20%; Guangzhou Insighter Biotechnology, Guangzhou, China), or antibiotic growth promoter (AGP; Carbadox, 50 ppm; added as Mecadox 10, Phibro Animal Health, Teaneck, NJ). The inclusion of BC-50 and Carbadox additives was according to manufacturer recommendations. The inclusion rate of BZA is a ratio of three-to-one equivalents of BC50. Each additive was mixed with ground corn up to the calculated content of corn in each diet. The additive-corn mix was then blended with the appropriate amount of each basal diet. The diets were provided as a mash. Titanium dioxide was included to determine apparent total tract digestibility (**ATTD**) of organic matter (**OM**), gross energy (**GE**), and crude protein (**CP**). The period 1 and period 2 basal diet samples were collected at mixing, ground to a 1-mm particle size (Eberbach E3503 Variable Speed Cutting Mill; Eberbach Corporation, Van Buren Charter Township, MI), and analyzed for dry matter (method 934.01; AOAC Int., 2006), OM (ash; method 942.05; AOAC Int., 2006), GE (Parr 6400 Automatic Isoperibol Calorimeter; Parr Instrument Company, Moline, IL), CP (rapid N cube; Elementar Americas, Ronkonkoma, NY), total amino acids (method 982.30; AOAC Int., 2006), and total calcium and phosphorus (method 985.01; AOAC Int., 2006) in five replicates per basal diet (Table 3). Period 1 and period 2 treatment diets also collected after mixing the additives and ground to a 1-mm particle size, as described earlier for basal diets.

**Table 1.** Composition and calculated nutrient content (as-fed basis) of period 1 treatment diets^a^

	Treatment^b^			
	NC	BZA	BC50	AGP
Ingredient, %				
Corn	55.69	55.09	55.49	55.46
Soybean meal	25	25	25	25
Soybean oil	2.50	2.50	2.50	2.50
Dried whey	8	8	8	8
Fish meal	5	5	5	5
L-Lysine·HCl	0.47	0.47	0.47	0.47
DL-Methionine	0.18	0.18	0.18	0.18
L-Threonine	0.18	0.18	0.18	0.18
L-Tryptophan	0.02	0.02	0.02	0.02
L-Valine	0.06	0.06	0.06	0.06
Limestone	1.00	1.00	1.00	1.00
Monocalcium phosphate	0.80	0.80	0.80	0.80
Salt	0.45	0.45	0.45	0.45
Vitamin premix^c^	0.30	0.30	0.30	0.30
Trace mineral premix^d^	0.15	0.15	0.15	0.15
Antibiotic^e^	—	—	—	0.23
Benzoic acid	—	0.60	—	—
BC-50	—	—	0.20	—
Titanium dioxide	0.20	0.20	0.20	0.20
Calculated nutrient composition				
ME, kcal/kg	3,436	3,415	3,429	3,428
Lactose, %	6.00	6.00	6.00	6.00
CP, %	21.4	21.3	21.3	21.3
SID Lys, %	1.40	1.40	1.40	1.40
SID Thr, %	0.85	0.85	0.85	0.85
SID Met, %	0.50	0.50	0.50	0.50
SID Met + Cys, %	0.77	0.77	0.77	0.77
SID Trp, %	0.24	0.24	0.24	0.24
SID Val, %	0.89	0.89	0.89	0.89
SID Ile, %	0.76	0.76	0.76	0.76
SID Lys:ME, g/Mcal	4.07	4.10	4.08	4.08
Total Ca, %	0.88	0.88	0.88	0.88
Total P, %	0.70	0.70	0.70	0.70
STTD P, %	0.46	0.46	0.46	0.46

^a^Period 1 treatment diets were fed for the first 7 d of the 21-d growth performance study.

^b^Benzoic acid, Benzocal-50, or Mecadox 10 are added to the base diet at the expense of corn.

^c^Vitamin premix provided the following per kg diet: vitamin A, 4950 IU; vitamin D3, 1980 IU; vitamin E, 53 IU; vitamin K, 4 mg; vitamin B12, 40 µg; riboflavin, 10 mg; niacin, 60 mg; and pantothenic acid, 33 mg.

^d^Trace mineral premix provided the following per kg diet: Fe (as ferrous sulfate), 109 mg; Cu (as copper sulfate), 16.5 mg; Zn (as zinc sulfate), 110 mg; Mn (as manganous oxide), 33 mg; I (as calcium iodate), 0.3 mg; and Se (as sodium selenite), 0.3 mg.

^e^Carbadox, 50 ppm (added as Mecadox 10, Phibro Animal Health).

Abbreviations: ME: metabolizable energy; SID: standardized ileal digestible; STTD: standardized total tract digestible.

**Table 2.** Composition and calculated nutrient content (as-fed basis) of period 2 treatment diets^a^

	Treatment^b^			
	NC	BZA	BC50	AGP
Ingredient, %				
Corn	58.94	58.34	58.74	58.71
Soybean meal	34	34	34	34
Soybean oil	2.50	2.50	2.50	2.50
L-Lysine·HCl	0.35	0.35	0.35	0.35
DL-Methionine	0.18	0.18	0.18	0.18
L-Threonine	0.13	0.13	0.13	0.13
Limestone	1.25	1.25	1.25	1.25
Monocalcium phosphate	1.40	1.40	1.40	1.40
Salt	0.60	0.60	0.60	0.60
Vitamin premix^c^	0.30	0.30	0.30	0.30
Trace mineral premix^d^	0.15	0.15	0.15	0.15
Antibiotic^e^	—	—	—	0.23
Benzoic acid	—	0.60	—	—
Benzocal-50	—	—	0.20	—
Titanium dioxide	0.20	0.20	0.20	0.20
Calculated nutrient composition				
ME, kcal/kg	3,381	3,361	3,374	3,373
CP, %	21.6	21.6	21.6	21.6
SID Lys, %	1.28	1.28	1.28	1.28
SID Thr, %	0.79	0.79	0.79	0.79
SID Met, %	0.47	0.47	0.47	0.47
SID Met + Cys, %	0.76	0.76	0.76	0.76
SID Trp, %	0.23	0.23	0.23	0.23
SID Val, %	0.84	0.84	0.84	0.84
SID Ile, %	0.78	0.78	0.78	0.78
SID Lys:ME, g/Mcal	3.79	3.81	3.79	3.79
Total Ca, %	0.84	0.84	0.84	0.84
Total P, %	0.70	0.70	0.70	0.70
STTD P, %	0.43	0.43	0.43	0.43

^a^Period 2 treatment diets were fed for the last 14 d of the 21-d growth performance study.

^b^Benzoic acid, Benzocal-50, or Mecadox 10 are added to the base diet at the expense of corn.

^c^Vitamin premix provided the following per kg diet: vitamin A, 4950 IU; vitamin D3, 1980 IU; vitamin E, 53 IU; vitamin K, 4 mg; vitamin B12, 40 µg; riboflavin, 10 mg; niacin, 60 mg; and pantothenic acid, 33 mg.

^d^Trace mineral premix provided the following per kg diet: Fe (as ferrous sulfate), 109 mg; Cu (as copper sulfate), 16.5 mg; Zn (as zinc sulfate), 110 mg; Mn (as manganous oxide), 33 mg; I (as calcium iodate), 0.3 mg; and Se (as sodium selenite), 0.3 mg.

^e^Carbadox, 50 ppm (added as Mecadox 10, Phibro Animal Health).

Abbreviations: ME: metabolizable energy; SID: standardized ileal digestible; STTD: standardized total tract digestible.

**Table 3.** Analyzed nutrient content (as-fed basis) of period 1 and period 2 basal diets^a,b^

Item	Period 1	Period 2
Dry matter, %	89.7	88.5
Gross energy, kcal/kg	3,967 (4,035)	3,925 (4,016)
Crude protein, %	21.2 (21.4)	21.4 (21.6)
Total Lys, %	1.61 (1.55)	1.54 (1.43)
Total Thr, %	0.96 (0.98)	0.95 (0.93)
Total Met, %	0.53 (0.54)	0.46 (0.51)
Total Met + Cys, %	0.84 (0.88)	0.80 (0.86)
Total Trp, %	0.24 (0.27)	0.23 (0.26)
Total Val, %	1.01 (1.03)	1.03 (0.98)
Total Ile, %	0.90 (0.87)	0.98 (0.89)
Total Ca, %	1.16 (0.91)	0.86 (0.88)
Total P, %	0.71 (0.70)	0.64 (0.70)

^a^Period 1 treatment diets were fed for the first 7 d of the 21-d growth performance study; period 2 treatment diets were fed for the last 14 d of the 21-d growth performance study.

^b^Values in parentheses indicate formulated values (NRC, 2012).

### Experimental Procedures and Sample Collection

Pens were assigned randomly to one of four dietary treatments according to BW (NC, BZA, BC50, or AGP; *n* = 8 pens per treatment). Treatment diets were fed over two periods. Pigs were provided with free access to fresh feed and water. Individual pig weights were measured at the start of the study and weekly thereafter for 3 weeks on a bench scale (Defender 5000; OHAUS, Parsippany, NJ). Feed addition was weighed was recorded daily; at the end of each week, remaining feed in each feeder was removed with a wet/dry vacuum, transferred into preweighed containers, weighed, and recorded on a bench scale. Spoiled feed was removed from feeders, weighed, and recorded. Pig morbidity, mortality, and removals were monitored daily and recorded.

Fecal samples were collected for ATTD of OM, GE, and CP analysis and fecal consistency scores over the last 3 d of each period. Pens were cleaned prior to and every 24 h thereafter for fecal sample collection. Pens were monitored hourly between 08:00 and 18:00 and fecal samples (combination of fresh fecal grab samples from the pen floor and rectal palpation) were collected, pooled per pen and period, and frozen at −20 °C. Feces that were obtained by rectal palpation only were assessed for fecal consistency. Fecal consistency from at least three pigs per pen was assessed in fresh feces obtained by rectal palpation at the end of period 1 and period 2 on a five-point scale: 1 = liquid; 2 = soft, unformed feces that does not retain shape; 3 = soft, moist feces that retains shape; 4 = firm, formed stool; and 5 = hard, dry pellet. Fecal consistency was scored by blinded individuals, and scores were averaged per pen.

At the end of each period, one pig per pen, closest to the mean pen BW, was euthanized by carbon dioxide asphyxiation (AVMA, 2020). After euthanasia, whole blood (intracardiac; collected into dipotassium EDTA vacutainers; BD, Franklin Lakes, NJ), jejunum (mid jejunum; sampled 100 cm from the ligament of Treitz), ileum (distal ileum; sampled 20 cm from the ileo-cecal junction), colon (center of spiral colon), and ileum and colon digesta were collected.

Plasma was separated from whole blood after centrifugation (3,000 × g for 5 min at room temperature), aliquoted, snap-frozen in liquid nitrogen, and stored at −80 °C. For intestinal morphometric analysis, 10-cm segments of jejunum and ileum were rinsed from debris with ice-cold phosphate-buffered saline (**PBS**) and placed immediately in neutral-buffered formalin. For cytokine and tight junction protein abundance in intestinal mucosa, 25-cm jejunum or ileum segments were opened longitudinally, pinned to a cork board, rinsed with PBS, scraped with a microscope slide to separate the mucosa from the underlying serosa, snap-frozen in liquid nitrogen, and stored at −80 °C; a 5-cm colon segment was opened longitudinally, rinsed with PBS, snap-frozen in liquid nitrogen, and stored at −80 °C. For digesta SCFA analysis, ileum and colon digesta were diluted with an equal weight of distilled water, vortexed, incubated on a platform rocker for 4 h at room temperature, and centrifuged at 3,000 × g for 10 min at room temperature. A 1-mL aliquot of the digesta supernatant was added to 200 µL meta-phosphoric acid (12.5% w/v) containing 2-ethylbutyric acid (8.6 mmol/L) as an internal standard and frozen at −20 °C.

### Sample Processing and Laboratory Analyses

#### Apparent total tract digestibility of organic matter, gross energy, and crude protein.

Fecal samples per pen were dried at 60 °C for 72 h, finely ground in a coffee grinder (DCG-12BC; Cuisinart, Stamford, CT), and passed through a 1-mm particle size sieve. Dried and ground treatment diets and fecal samples were analyzed for OM (method 942.05; AOAC Int., 2006), GE (Parr 6400 Automatic Isoperibol Calorimeter), and CP (rapid N cube). Titanium content in treatment diet and fecal dry ash was analyzed to Myers et al. (2004) with minor modifications.

#### Jejunum and ileum morphology.

Morphometric analysis was performed on formalin-fixed jejunum and ileum samples that were embedded in paraffin, sectioned at 4 µm, stained with hematoxylin and eosin, and imaged using an VS200 Research Slide Scanner (Olympus Life Sciences, Tokyo, Japan). Villus height and crypt depth were determined in approximately 10 well-oriented, intact, and symmetrical villi and adjacent crypts per jejunum and ileum using QuPath image analysis software (version 0.4.3; Bankhead et al., 2017). Villus height was measured from the villus tip to the crypt-villus junction, and crypt depth was measured from the crypt-villus junction to the muscularis mucosae.

#### Plasma glucose, nonesterified fatty acids, and urea.

Plasma glucose and nonesterified fatty acid (**NEFA**) concentrations were measured enzymatically with commercially available kits by endpoint analysis (glucose, LabAssay Glucose; NEFA, LabAssay NEFA; Wako Chemicals USA, Richmond, VA). Plasma urea nitrogen (**PUN**) concentration was measured enzymatically with a commercial kit by kinetic analysis (Urea Nitrogen (BUN) Liqui-UV (Rate); Stanbio Laboratory, Boerne, TX). Absorbance measurements were quantified with a Synergy 4 multimode plate reader (BioTek Instruments, Winooski, VT).

#### Plasma amino acids.

Plasma samples (100 µL) were deproteinized with an equal volume of trichloroacetic acid (TCA; 10% w/v). After centrifugation (12,000 × g for 20 min at 4 °C), clarified plasma supernatant (10 µL) and norvaline internal standard (10 µL; 250 µmol/L) or an amino acid standard (containing 250 µmol/L of all amino acids, including citrulline, ornithine, taurine, and norvaline internal standard, and 125 µmol/L cystine) were alkalinized with sodium hydroxide (10 µL; 0.35 mol/L) and sodium tetraborate (50 µL; 125 mmol/L, pH 9.5). Amino acids were derivatized with 6-aminoquinolyl-N-hydroxysuccinimidyl carbamate (**AQC**; 20 µL; 3 mg/mL in acetonitrile; Cayman Chemical Company, Ann Arbor, MI) for 1 min at room temperature and 10 min at 55 °C. Derivatized amino acids were separated on a C18 analytical column (Waters Acquity UPLC BEH C18, 1.7 µm × 150 mm) on an Acquity UPLC H-Class PLUS System equipped with a quaternary pump, fluorescence detector, and Empower 3 software (Waters, Milford, MA). Mobile phases were 0.1% formic acid in water (mobile phase A; filtered through a 0.20-µm cellulose acetate membrane) and acetonitrile (mobile phase B). Separation was performed at a flow rate of 300 µL/min with the following gradient: 0 min, 0% B; 0–8 min, 4% B; 8–12 min, 10% B; 12–18 min, 20% B; 18–18.5 min, 80% B; 18.5–20 min, 80% B; 20–20.5 min, 0% B; 20.5–24 min, 0% B. All gradient changes were linear. The sample manager temperature was 20 °C, and the column temperature was maintained at 45 °C. The injection volume was 1 µL. The excitation wavelength was set at 266 nm; the emission wavelength of 473 nm was monitored at 10 Hz.

Plasma cysteine and homocysteine concentrations were determined separately from other amino acids due to their tendency to form mixed and protein disulfides in oxidizing conditions (Toyo’oka, 2009). Briefly, plasma samples (50 µL) and 2-mercaptopropionlylglycine internal standard (50 µL; 125 µmol/L) were reduced with tris-(2-carboxyethyl)-phosphine hydrochloride (20 µL; 30 mmol/L) for 15 min at room temperature. Reduced plasma samples were subsequently deproteinized with TCA (80 µL; 10% w/v) and centrifuged (12,000 × g for 20 min at 4 °C). Clarified plasma supernatant (80 µL) was alkalinized with sodium hydroxide (20 µL; 0.50 mol/L), derivatized with 4-fluoro-7-aminosulfonylbenzofurazan (**ABD-F**; 100 µL; 1 mg/mL in 125 mmol/L sodium tetraborate, pH 9.5) for 15 min at 50 °C and quenched with hydrochloric acid (50 µL; 1 mol/L). Cysteine- and homocysteine-ABD derivatives were analyzed by reverse phase high performance liquid chromatography according to Rudar et al. (2023).

#### Distal ileum and colon digesta SCFA.

 SCFA analysis was performed with an Agilent 8890B gas chromatograph (GC; Agilent Technologies, Santa Clara, CA). SCFAs were resolved with a J&W DB-FATWAX Ultra Inert column (length, 30 m; internal diameter, 0.25 mm; film thickness, 0.25 µm; Agilent Technologies) and detected by flame ionization. The following GC parameters were used: carrier gas: nitrogen, 18.32 mL/min, constant flow mode; inlet: split/splitless, 250 °C, 30:1 split ratio; oven: 162 °C, isothermal; flame ionization detector: 250 °C, 30 mL hydrogen/min, 400 mL air/min, 25 mL make-up gas/min; injection volume: 0.1 µL. SCFA concentrations were corrected for dilution.

#### Plasma, jejunum, ileum, and colon cytokine abundance.

Jejunum, ileum, and colon tissue (100 mg) was homogenized in lysis buffer on ice and centrifuged at 12,000 × g for 20 min at 4 °C (Chen et al., 2020). Protein concentrations in the clarified lysate were determined by the bicinchoninic acid assay. Plasma (100 µL) was analyzed for tumor necrosis factor-α (**TNF-α**; porcine TNF-alpha DuoSet ELISA kit, R&D Systems, Minneapolis, MN); tissue lysate (150 µg protein) was analyzed for TNF-α, interleukin-1β (**IL-1β**; porcine IL-1 beta/IL-1F2 DuoSet ELISA kit, R&D Systems), and interleukin-8 (**IL-8**; porcine IL-8/CXCL8 DuoSet ELISA, R&D Systems). Absorbance was measured at 450 nm and corrected at 550 nm. Intra-assay CV was 4.2%, 1.7%, and 1.8% for TNF-α, IL-1β, and IL-8, respectively. Inter-assay CV was 6.7%, 11.6%, and 10.3% for TNF-α, IL-1β, and IL-8, respectively.

#### Immunoblot analysis.

Jejunum, ileum, and colon tissue was homogenized in 15 volumes of RIPA lysis buffer. The clarified homogenate was subsampled to determine protein concentration by the bicinchoninic acid assay, and protein was denatured for 10 min at 70 °C after addition of Laemmli loading buffer. Lysates were subject to SDS-PAGE analysis; 40 μg protein was loaded per lane. Following electrophoresis, proteins were transferred to low-fluorescence PVDF membranes, blocked with 5% w/v nonfat dry milk in PBS-T, and incubated with primary antibody (diluted in 5% w/v BSA in PBS-T) and secondary antibodies (diluted in 5% w/v nonfat dry milk in PBS-T). The following primary antibodies were used: high mobility group box 1 (HMGB1; 1:5,000; RRID: AB_2544854; PA5-27378, Thermo Fisher); claudin 3 (CLDN3; 1:20,000; RRID: AB_10981317; PA5-16867, Thermo Fisher); and claudin 4 (CLDN4; 1:1,000; RRID: AB_2533096; 32-9400, Thermo Fisher). The dilution of the corresponding secondary antibody (HRP-conjugated anti-mouse IgG; 170-6515, Bio-Rad, Hercules, CA; HRP-conjugated anti-rabbit IgG; 170-6516, Bio-Rad) was 1:20,000. Immunoreactivity was visualized with chemiluminescence (ChemiDoc XRS+ imager, Bio-Rad). Target protein abundance was normalized against total protein transferred onto the membrane by fluorescent modification of tryptophan residues on proteins with 2,2,2-trichloroethanol (Chopra et al., 2019). Target protein abundance across blots was normalized to a pooled sample analyzed on each blot within each tissue. Volumetric analysis of target protein bands and total lane protein was performed with Image Lab software (version 6.0; Bio-Rad).

#### Calculations and statistical analyses.

Growth performance (average daily gain, average daily feed intake, and gain-to-feed) were calculated with initial pen weight, pen weight at the end of period 1, pen weight at the end of period 2, and total pig days (pigs × days in period). Time and pig weight for any pig removed from the study were recorded immediately upon removal to account for incomplete animal data in growth performance calculations. Apparent total tract digestibility was calculated according to Stein et al. (2007).

Data were analyzed with the generalized linear mixed model procedure of SAS (SAS 9.4, SAS Institute, Cary, NC). Growth performance data were analyzed by 1-factor ANOVA per period; diet was considered the main effect; pen and block were considered random effects. Fecal consistency scores, ATTD of OM, GE, and CP, plasma amino acid concentrations, digesta SCFA concentrations, plasma and tissue cytokine concentrations, and protein abundance data were analyzed by 2-factor ANOVA; diet (NC, BZA, BC50, and AGP) and period were considered the main effects; pen (fecal consistency score, ATTD), pig (plasma glucose, plasma NEFA, PUN, amino acids, digesta SCFAs, and tissue cytokine and protein abundance), and block were considered random effects. Normality of residuals was assessed with the Shapiro–Wilk test statistic. Data are presented as least-squares means ± standard error of the mean. Tissue cytokine abundance was log transformed to normalize residual distribution; least-square means and standard error of the mean were back transformed for data presentation. Differences among treatment diets were determined with a Tukey post-hoc test. Statistical significance and tendency were considered at *P* ≤ 0.05 and *P* ≤ 0.10, respectively.

## RESULTS

Diet analysis indicated that the content of gross energy, crude protein, amino acids, and minerals were largely consistent with the calculated nutrient content of each experimental diet (Table 3). Thus, any differences in growth performance, nutrient digestibility, or physiological response are attributable to the tested additives in each diet. Two pigs (one from the BZA group and one from the BC50 group) were removed from the study due to lameness; one pig from the BZA group was removed from the study due to *Streptococcus suis* septicemia. Pig removal and mortality rates were not different among treatments (*P* > 0.10).

### Growth Performance

Initial BW and BW after period 1 and period 2 did not differ among groups (Table 4; *P* > 0.10). Period 1 ADG and ADFI were greater in the AGP group compared to the NC group, BZA, and BC50 groups (*P* < 0.05), whereas ADG and ADFI were not different among the NC, BZA, and BC50 groups (*P* > 0.10). Feed efficiency (gain: feed, G: F) was similar among groups (*P* > 0.10). Period 2 ADG was greatest in the AGP group, intermediate in the BC50 group, and lowest in the NC and BZA groups (*P* < 0.01). Period 2 ADFI was greatest in the AGP group, intermediate in the BZA and BC50 groups, and lowest in the NC group (*P* < 0.05). However, feed efficiency was lower in the BZA group compared to the NC, BC50, and AGP groups (*P* < 0.05). Overall, ADG was greatest in the AGP group (*P* < 0.01), ADFI was greatest in the AGP group, intermediate in the BZA group, and lowest in the NC and BC50 groups (*P* < 0.01), and feed efficiency was lowest in the BZA group (*P* < 0.01).

**Table 4.** Effect of dietary treatment and period on starter pig performance

	Treatment^a^					
Item	NC	BZA	BC50	AGP	SEM^b^	*P*-value
Initial BW, kg	8.53	9.11	8.55	8.71	0.41	0.74
Period 1 BW, kg	9.77	10.41	9.87	10.48	0.41	0.52
Period 2 BW, kg	17.14	17.81	17.78	19.14	0.61	0.15
Period 1^a^						
ADG, g/d	178^†^	179^†^	185^†^	253^^*^^	17	0.012
ADFI, g/d	295^†^	310^†^	300^†^	373^^*^^	21	0.006
G:F, g/g	0.60	0.58	0.60	0.68	0.05	0.33
Period 2^a^						
ADG, g/d	534^†^	533^†^	562^*,†^	620^*^	31	0.009
ADFI, g/d	801^†^	859^*,†^	846^*,†^	940^*^	27	0.010
G:F, g/g	0.67^*^	0.62^†^	0.66^*^	0.66^*^	0.03	0.04
Period 1 + Period 2^a^						
ADG, g/d	401^†^	398^†^	420^†^	482^*^	23	0.002
ADFI, g/d	612^†^	648^*,†^	639^†^	728^*^	23	0.007
G:F, g/g	0.66^*^	0.62^†^	0.66^*^	0.66^*^	0.03	0.007

^a^Pigs were fed experimental diets at weaning (28 d age) for 21 d. Treatments: NC: negative control (no additive); BZA: benzoic acid (0.60%); BC50: Benzocal-50 (0.20%); AGP: antibiotic growth promoter (Carbadox, 50 ppm). Period: period 1 = 7 d; period 2 = 14 d.

^b^Maximum value for the standard error of the mean.

^*,†^Means without a common superscript differ after Tukey multiple comparison test, *P* < 0.05.

Abbreviations: G:F: gain-to-feed.

### Apparent Total Tract Digestibility of Organic Matter, Gross Energy, and Crude Protein

Apparent total tract digestibility of OM and GE was greatest in the AGP group, intermediate in the BZA group, and lowest in the NC and BC50 groups (Table 5; *P* < 0.01). Apparent total tract digestibility of CP was greatest in the AGP group, intermediate in the NC and BZA groups, and lowest in the BC50 group (*P* < 0.01). Apparent total tract digestibility of OM was greater in period 1 (*P* = 0.007), ATTD GE was not different between periods (*P* > 0.10), and ATTD CP was greater in period 2 (*P* < 0.001). There were no interactions between treatment and period on ATTD OM, ATTD GE, or ATTD CP (*P* > 0.10).

**Table 5.** Effect of dietary treatment and period on apparent total tract digestibility of organic matter, gross energy, and crude protein and fecal consistency score

	Treatment^a^				Period			*P*-value		
Item^b^	NC	BZA	BC50	AGP	1	2	SEM^c^	Treatment	Period	Treatment × period
ATTD OM, %	81.6^†^	82.6^*,†^	80.9^†^	84.4^*^	83.1	81.6	0.9	0.004	0.007	0.15
ATTD GE, %	79.0^†^	80.1^*,†^	78.2^†^	82.3^*^	80.1	79.7	0.9	0.001	0.41	0.20
ATTD CP, %	74.0^†,‡^	75.7^*,†^	71.4^‡^	78.4^*^	73.1	76.6	2.1	<0.001	<0.001	0.17
Fecal consistency^b^	2.41^†^	2.79^*,†^	2.53^†^	3.09^*^	2.75	2.66	0.15	<0.001	0.34	0.44

^a^Pigs were fed experimental diets at weaning (28 d age) for 21 d. Treatments: NC: negative control (no additive); BZA: benzoic acid (0.60%); BC50: Benzocal-50 (0.20%); AGP: antibiotic growth promoter (Carbadox, 50 ppm). Period: period 1 = 7 d; period 2 = 14 d.

^b^Maximum value for the standard error of the mean.

^c^Fecal consistency score: 1 = liquid; 2 = soft, unformed feces that does not retain shape; 3 = soft, moist feces that retains shape; 4 = firm, formed stool; and 5 = hard, dry pellet.

^*,†,‡^Means without a common superscript differ after Tukey multiple comparison test, *P* < 0.05.

### Fecal Consistency

Fecal consistency was greatest in the AGP group, intermediate in the BZA group, and lowest in the BC50 and NC groups (Table 5; *P* < 0.001). There was no effect of period or interaction between treatment and period on fecal consistency (*P* > 0.10).

### Jejunum and Ileum Morphology

Both jejunum and ileum villus height and crypt depth increased from period 1 to period 2 (Table 6; *P* < 0.01), but there was no effect of treatment or interaction between treatment and period on these parameters (*P* > 0.10). The villus height to crypt depth ratio in the ileum tended to increase from period 1 to period 2 (*P* = 0.09).

**Table 6.** Effect of dietary treatment and period on jejunum and ileum histomorphology

	Treatment^a^				Period				*P*-value	
Item^b^	NC	BZA	BC50	AGP	1	2	SEM^c^	Treatment	Period	Treatment × period
Jejunum										
Villus height, µm	478	477	538	502	461	536	37	0.28	0.003	0.20
Crypt depth, µm	227	229	236	213	210	243	18	0.17	<0.001	0.12
VH:CD ratio	2.14	2.09	2.32	2.37	2.24	2.21	0.14	0.29	0.77	0.43
Ileum										
Villus height, µm	326	324	354	320	292	369	37	0.20	<0.001	0.22
Crypt depth, µm	211	210	205	189	187	221	7	0.11	<0.001	0.44
VH:CD ratio	1.54	1.57	1.74	1.72	1.58	1.71	0.17	0.20	0.09	0.20

^a^Pigs were fed experimental diets at weaning (28 d age) for 21 d. Treatments: NC: negative control (no additive); BZA: benzoic acid (0.60%); BC50: Benzocal-50 (0.20%); AGP: antibiotic growth promoter (Carbadox, 50 ppm). Period: period 1 = 7 d; period 2 = 14 d.

^b^Maximum value for the standard error of the mean.

^*,†,^Means without a common superscript differ after Tukey multiple comparison test, *P* < 0.05.

Abbreviations: CD: crypt depth; VH: villus height.

### Plasma Glucose, Non-Esterified Fatty Acids, and Urea

Plasma glucose concentrations were not different among diets (Table 7; *P* > 0.10) but were greater in period 1 than in period 2 (*P* > 0.01). Plasma NEFA concentrations were not different among diets or between period 1 and period 2 (*P* > 0.10). Plasma urea concentrations were greatest in the AGP group, intermediate in the NC and BC50 groups, and lowest in the BZA group (*P* < 0.05) and increased from period 1 to period 2 (*P* < 0.001). There were no interactions between treatment and period on plasma glucose, NEFA, or urea concentrations (*P* > 0.10).

**Table 7.** Effect of dietary treatment and period on plasma glucose (mg/dL), non-esterified fatty acid (µmol/L), urea (mg/dL), and amino acid concentrations (µmol/L)

	Treatment^a^				Period			*P*-value		
Item	NC	BZA	BC50	AGP	1	2	SEM^c^	Treatment	Period	Treatment × period
Glucose	147	142	149	142	167	123	14	0.96	<0.001	0.90
NEFA	90	101	86	104	87	103	16	0.68	0.18	0.52
PUN	9.5^*,†^	8.2^†^	9.5^*,†^	11.4^*^	8.0	12.3	1.1	0.08	<0.001	0.27
NEAA^c^										
Ala	358	364	367	359	430	294	15	0.98	<0.001	0.88
Arg	89	108	91	101	91	104	12	0.18	0.06	0.92
Asn	59	72	60	70	64	66	5	0.16	0.64	0.72
Asp	33	37	30	34	38	29	3	0.23	<0.001	0.74
Cys^d^	183^†^	199^*,†^	190^*,†^	208^*^	190	200	8	0.004	0.04	0.34
Glu	236	228	236	220	276	184	40	0.90	<0.001	0.39
Gln	314	286	285	317	315	287	26	0.25	0.06	0.09
Gly	616^*^	492^†^	608^*,†^	684^*^	606	594	45	0.001	0.69	0.94
Pro	159	173	164	175	170	165	8	0.49	0.56	0.90
Ser	97^†^	99^*,†^	98^†^	119^*^	98	108	6	0.02	0.09	0.94
Tyr	121	136	114	128	107	142	9	0.15	<0.001	0.81
EAA^c^										
His	45	52	46	49	49	47	4	0.21	0.40	0.19
Ile	82	92	79	90	90	82	4	0.06	0.05	0.68
Leu	106	124	108	125	114	117	6	0.04	0.63	0.36
Lys	115	151	131	138	137	130	22	0.10	0.53	0.74
Met	38	44	43	43	39	45	5	0.40	0.06	0.76
Phe	48^†^	56^*^	49^*,†^	53^*,†^	53	50	2	0.02	0.12	0.56
Thr	121^†^	171^*^	135^*,†^	153^*,†^	138	152	12	0.03	0.23	0.41
Val	108	129	104	128	107	128	10	0.03	0.006	0.17
Other AA^c^										
Cit	28^*,†^	29^*,†^	25^†^	32^*^	27	30	2	0.02	0.04	0.97
Hcy^d^	27	25	22	31	22	31	3	0.19	0.002	0.65
Orn	66	72	64	70	63	73	5	0.66	0.04	0.91
Tau	125	124	106	148	102	149	26	0.30	0.003	0.10
NEAA	2,265	2,191	2,245	2,414	2,385	2,172	79	0.24	0.01	0.85
EAA	662^†^	818^†^	692^*,†^	778^*,†^	731	744	36	0.01	0.71	0.39
BCAA	296	345	291	343	311	327	19	0.03	0.33	0.33

^a^Pigs were fed experimental diets at weaning (28 d age) for 21 d. Treatments: NC: negative control (no additive); BZA: benzoic acid (0.60%); BC50: Benzocal-50 (0.20%); AGP: antibiotic growth promoter (Carbadox, 50 ppm). Period: period 1 = 7 d; period 2 = 14 d.

^b^Maximum value for the standard error of the mean.

^c^Amino acids were analyzed in plasma following deproteinization and derivatization with AQC; tryptophan-AQC derivative was not able to be detected by fluorescence.

^d^Cys and Hcy were analyzed in plasma following disulfide bond reduction, deproteinization, and derivatization with 4-(aminosulfonyl)-7-fluoro-2,1,3-benzoxadiazole.

^*,†^Means without a common superscript differ after Tukey multiple comparison test, *P* < 0.05.

Abbreviations: AA: amino acid; Ala: alanine; AQC: 6-aminoquinolyl-*N*-hydroxysuccinimidyl carbamate; Arg: arginine; Asn: asparagine; BCAA: branched-chain amino acid; Cit: citrulline; Cys: cysteine; EAA: essential amino acid; Glu: glutamate; Gln: glutamine; Gly: glycine; His: histidine; Hcy: homocysteine; Ile: isoleucine; Leu: leucine; Lys: lysine; Met: methionine; NEAA: non-essential amino acid; NEFA: non-esterified fatty acid; Orn: ornithine; Phe: phenylalanine; Pro: proline; PUN: plasma urea nitrogen; Ser: serine; Tau: taurine; Thr: threonine; Tyr: tyrosine; Val: valine.

### Plasma Amino Acids

Plasma glycine concentrations were lower in the BZA group compared to the NC and AGP groups, whereas glycine in the BC50 group was intermediate between the BZA, NC, and AGP groups (Table 7; *P* < 0.01). Plasma alanine, aspartate, and glutamate concentrations were not different among diets (*P* > 0.10) but declined from period 1 to period 2 (*P* < 0.001). There were no interactions between treatment and period on any amino acid (*P* > 0.10).

### Distal Ileum and Colon Digesta SCFA

There was no effect of diet, period, or interaction between diet and period on acetate concentrations in the distal ileum (*P* > 0.10). Colon propionate, butyrate, and total SCFA concentrations were not different among groups (Table 8; *P* > 0.10), whereas colon acetate and valerate tended to be greater in the BZA group (*P* < 0.10). Both colon acetate and total SCFA concentrations declined from period 1 to period 2 (*P* < 0.001). The ratio of acetate, propionate, and butyrate to total SCFA was not different among diets and between period 1 and period 2 (*P* > 0.10). There were no interactions between treatment and period for individual SCFA, total SCFA, or SCFA ratios (*P* > 0.10).

**Table 8.** Effect of dietary treatment and period on colon digesta short-chain fatty acid concentrations (mmol/L)

	Treatment^a^				Period			*P*-value		
Item	NC	BZA	BC50	AGP	1	2	SEM^3^	Treatment	Period	Treatment × Period
Acetate	39.5	48.8	44.8	41.9	49.8	37.6	8.2	0.10	< 0.001	0.26
Propionate	27.4	33.9	28.5	28.9	33.1	26.2	5.6	0.19	0.004	0.54
Butyrate	12.5	15.8	15.0	13.4	15.3	13.0	2.7	0.38	0.13	0.31
Valerate	3.5	4.7	4.2	2.8	4.2	3.4	0.6	0.07	0.12	0.15
Total SCFA	82.8	103.2	92.5	86.9	102.4	80.3	16.7	0.13	< 0.001	0.29
Acetate acid: Total SCFA	0.48	0.48	0.49	0.50	0.49	0.48	0.01	0.72	0.43	0.45
Propionate: Total SCFA	0.33	0.33	0.31	0.32	0.32	0.32	0.01	0.31	0.71	0.16
Butyrate: Total SCFA	0.14	0.15	0.16	0.15	0.15	0.16	0.01	0.33	0.09	0.40

^a^Pigs were fed experimental diets at weaning (28 d age) for 21 d. Treatments: NC: negative control (no additive); BZA, : benzoic acid (0.60%); BC50: Benzocal-50 (0.20%); AGP: antibiotic growth promoter (Carbadox, 50 ppm). Period: period 1 = 7 d; period 2 = 14 d.

^2^Maximum value for the standard error of the mean.

^*,†^Means without a common superscript differ after Tukey multiple comparison test, *P* < 0.05.

### Plasma, Jejunum, Ileum, and Colon Cytokine Abundance

Plasma TNF-α was not different among groups (*P* > 0.10). While jejunum proinflammatory cytokine abundance was also not different among groups (*P* > 0.10), TNF-α, IL-1β, and IL-8 all declined from period 1 to period 2 (*P* < 0.05). Ileum TNF-α abundance was greatest in the AGP group, intermediate in the NC and BZA groups, and lowest in the BC50 group (Table 9; *P* < 0.05). Colon TNF-α abundance was similarly greatest in the AGP group, intermediate in the NC and BC50 groups, and lowest in the BZA group (*P* < 0.05). Ileum IL-1β abundance was not different among groups (*P* > 0.10), whereas colon IL-1β abundance was greatest in the BZA group, intermediate in the NC and BC50 groups, and lowest in the AGP group (*P* < 0.05). Ileum IL-8 abundance tended to be greater in the BZA and BC50 groups than the NC and AGP groups (*P* = 0.07), whereas colon IL-8 abundance was greatest in the BZA group, intermediate in the NC and BC50 groups, and lowest in the AGP group (*P* < 0.05).

**Table 9.** Effect of dietary treatment and period on plasma tumor necrosis factor-α concentration (pg/mL) and jejunum, ileum, and colon tumor necrosis factor-α, interleukin-1β, and interleukin-8 abundances (pg/mg protein)

	Treatment^a^				Period^a^			*P*-value		
Item	NC	BZA	BC50	AGP	1	2	SEM^b^	Treatment	Period	Treatment × period
Plasma TNF-α	179	138	148	115	167	123	53	0.77	0.31	0.54
Jejunum										
TNF-α	9.2	7.6	7.2	8.1	8.8	7.3	0.8	0.22	0.03	0.53
IL-1β	78.6	66.1	60.0	53.4	78.7	51.8	15.2	0.21	0.002	0.91
IL-8	619	605	697	646	716	574	44	0.40	<0.001	0.15
Ileum										
TNF-α	25.1^*,†^	21.1^*,†^	19.3^†^	35.1^*^	24.3	24.7	7.3	0.04	0.92	0.88
IL-1β	110	108	119	96	115	102	12	0.48	0.21	0.62
IL-8	1,072	1,422	1,300	1,071	1,197	1,217	154	0.07	0.85	0.34
Colon										
TNF-α	38.6^*,†^	20.4^†^	24.6^*,†^	43.5^*^	25.8	35.6	7.8	0.01	0.07	0.74
IL-1β	139^*,†^	171^*^	134^*,†^	86^†^	147	112	32	0.02	0.08	0.97
IL-8	267^*,†^	314^*^	258^*,†^	186^†^	222	287	63	0.03	0.04	0.99

^a^Pigs were fed experimental diets at weaning (28 d age) for 21 d. Treatments: NC: negative control (no additive); BZA: benzoic acid (0.60%); BC50: Benzocal-50 (0.20%); AGP: antibiotic growth promoter (Carbadox, 50 ppm). Period: period 1 = 7 d; period 2 = 14 d.

^b^Maximum value for the standard error of the mean.

^*,†^Means without a common superscript differ after Tukey multiple comparison test, *P* < 0.05.

### Immunoblot Analysis

The total abundance of tight junction proteins CLDN3 and CLDN4 in the jejunum, ileum, and colon did not differ among treatment groups (Table 10; *P* > 0.10). While the abundance of jejunum CLDN4 declined from period 1 to period 2 (*P* = 0.003), jejunum CLDN3 did not change between periods (*P* > 0.10). Conversely, ileum CLDN3, but not CLDN4, abundance increased from period 1 to period 2 (*P* = 0.001). In colon, CLDN3 abundance increased from period 1 to period 2 (*P* = 0.02) and CLDN4 abundance tended to increase from period 1 to period 2 (*P* = 0.09). The abundance of HMGB1, an upstream mediator of cytokine secretion in immune cells, in the jejunum and ileum mucosa was not affected by treatment (*P* > 0.10). Ileum and colon abundance of HMGB1 declined from period 1 to period 2 (*P* < 0.05). There were no interactions between treatment and period on jejunum, ileum, or colon protein abundance.

**Table 10.** Effect of dietary treatment and period on jejunum, ileum, and colon abundance (AU) of high-mobility group box 1, claudin 3, and claudin 4

	Treatment^a^				Period			*P*-value		
Item	NC	BZA	BC50	AGP	1	2	SEM^b^	Treatment	Period	Treatment × period
Jejunum^c^										
HMGB1	0.94	0.89	0.83	0.85	0.88	0.88	0.08	0.54	0.99	0.25
CLND3	1.20	1.37	1.22	1.10	1.22	1.23	0.17	0.54	0.98	0.92
CLDN4	1.50	1.62	1.98	1.84	2.13	1.33	0.28	0.52	0.003	0.78
Ileum^d^										
HMGB1	1.13	0.98	1.11	0.99	1.15	0.95	0.10	0.45	0.02	0.38
CLND3	1.04	1.10	1.37	1.12	0.85	1.47	0.18	0.59	< 0.001	0.85
CLDN4	1.66	1.79	2.15	1.78	1.97	1.72	0.41	0.66	0.40	0.74
Colon^e^										
HMGB1	1.13	1.06	1.07	1.12	1.21	0.98	0.12	0.96	0.04	0.61
CLND3	1.66	1.08	1.29	1.92	0.97	2.00	0.50	0.50	0.02	0.51
CLDN4	1.79	1.63	1.43	1.01	0.95	1.97	0.72	0.83	0.09	0.87

^a^Pigs were fed experimental diets at weaning (28 d age) for 21 d. Treatments: NC: negative control (no additive); BZA: benzoic acid (0.60%); BC50: Benzocal-50 (0.20%); AGP: antibiotic growth promoter (Carbadox, 50 ppm). Period: period 1 = 7 d; period 2 = 14 d.

^b^Maximum value for the standard error of the mean.

^c^Representative blot images for jejunum CLDN3, CLDN4, and HMGB1 are provided in Supplementary Figure S1.

^d^Representative blot images for ileum CLDN3, CLDN4, and HMGB1 are provided in Supplementary Figure S2.

^e^Representative blot images for colon CLDN3, CLDN4, and HMGB1 are provided in Supplementary Figure S3.

^*,†^Means without a common superscript differ after Tukey multiple comparison test, *P* < 0.05.

## DISCUSSION

Weaning disrupts intestinal architecture and impairs nutrient utilization, mucosal immunity, and gut barrier capacity, limiting growth performance and increasing diarrhea incidence in pigs (Pluske et al., 2018). Dietary supplementation with free benzoic as an alternative to antibiotic growth promoters or zinc oxide is thought to reduce gastric pH, improve protein digestion, and limit the flow of undigested protein to the hindgut in newly weaned pigs (Heo et al., 2013). The ostensible benefits of benzoic acid in turn promote growth performance in starter pigs (Chen et al., 2017; Kiarie et al., 2018; Choi et al., 2023). However, rapid absorption of benzoic acid limits its ability to exert its potential antimicrobial and immunomodulatory effects beyond the stomach and proximal small intestine (Kristensen et al., 2009). In the current study, we investigated the effects of BC-50, a protected preparation of benzoic acid targeted for release in the mid and distal segments of the gut, on growth performance, nutrient digestibility, plasma metabolites, and gut health indices in starter pigs weaned at 28 d age. Most farms in the U.S. wean pigs between 19 and 22 d age, whereas other regions, such as the Europen Union, routinely wean older pigs at about 28 d age. Despite differences in weaning age, growth performance, gut structure (e.g., morphology), and gut function (e.g., barrier capacity, digestive enzyme activity) are impaired after weaning at both 21 d and at 28 d age; however, the extent that these parameters decline is greater in pigs weaned at 21 d age than in pigs weaned at 28 d age (Tsukahara et al., 2016; Faccin et al., 2020a). Moreover, a recent comprehensive study reported that in-feed antibiotics increase ADFI and G:F in the nursery, and overall wean-to-market growth performance, in pigs independent of weaning age (Faccin et al., 2020b). This suggests that alternative nursery diet additives, including free and protected benzoic acid, could function comparably. Thus, the present findings should be broadly applicable to pigs at weaning.

The AGP group consistently exhibited greater ADG and ADFI than the NC and BZA groups across the two feeding periods. Overall growth performance of the AGP group was greater than the BC50 group, but there was no significant difference in ADG and ADFI between the AGP and BC50 groups in period 2, which accounts for the bulk of BW gain and feed intake in this study. Conversely, benzoic acid failed to enhance starter pig growth performance in this study compared to NC group. Although several studies have reported an immediate benefit of benzoic acid on starter pig growth performance in the immediate postweaning period in pigs weaned at 21 d age (Chen et al., 2017; Kiarie et al., 2018) and at 28 d age (Torrallardona et al., 2007), other studies have report smaller effects in the same period in pigs weaned at 21 d age and supplemented BZA (Graber et al., 2012; Choi et al., 2023) or in pigs weaned at 28 d age and supplemented a protected organic acid blend containing benzoic acid (Correa et al., 2021). Instead, these studies indicate that most benefit of benzoic acid supplementation in starter pigs appears to occur later in the nursery period.

Feed efficiency was similar among the NC, BC50, and AGP groups but significantly lower in the BZA group. Considering that benzoic acid is excreted in urine as its glycine conjugate, hippuric acid, it is possible that benzoic acid feeding increased demand for glycine and limited glycine availability for lean protein accretion and decreased glycine catabolism to urea. This is consistent with the reduction in plasma glycine and urea concentrations in the BZA group; since protected benzoic acid was fed at a ratio of one to three equivalents of free benzoic acid, the reduction in plasma glycine and urea concentrations in the BC50 group was not as marked. The BZA group may have compensated for lower glycine availability by increasing feed intake, but this did not correspond to increased BW gain. Moreover, plasma alanine, aspartate, and glutamate, key intermediates in amino acid nitrogen metabolism, declined from period 1 to period 2. This decline could reflect heightened demand for nonessential amino acid nitrogen in pigs recovered from the immediate postweaning growth check. Increased glycine utilization for benzoic acid conjugation and excretion may have thus exacerbated the lower availability of nonessential amino acids for lean protein accretion through additional demand of nonessential amino acid nitrogen for glycine synthesis. This ultimately may have negatively affected feed efficiency in the BZA group. Although total CP intake was unlikely to limit growth performance, low bioavailability of individual nonessential amino acids, such as glycine, could reduce BW gain and feed efficiency in pigs (Powell et al., 2011).

Despite similar ATTD of OM and GE in the BZA and BC50 groups, lower ATTD of CP in the BC50 group compared to the BZA group did not translate to impaired growth performance. The reduction in ATTD of CP in pigs supplemented protected compared to BZA is likely due less organic acid-mediated gastric acidification, pepsin activation, and protein hydrolysis in the proximal gut (Suiryanrayna and Ramana, 2015). This result agrees with other studies that demonstrated improved ATTD of CP in response to benzoic acid supplementation in pigs weaned at 21 d age (Kiarie et al., 2018) and at 28 d age (Kluge et al., 2006; Diao et al., 2016). However, an improvement in ATTD of CP in response to benzoic acid has not been a consistent observation (Murphy et al., 2011; Humphrey et al., 2022) and likely reflects less external need to acidify gastric contents in older pigs. Although greater ATTD of OM, GE, and CP in the AGP group compared to the NC group could contribute to the increase in growth performance, these did not correspond to differences in jejunum or ileum villus height and crypt depth, an index of intestinal digestive and absorptive capacity and the extent of intestinal maturity (Pluske et al., 2018), or to substantial differences in plasma glucose, NEFA, and amino acid concentrations among groups, a broad readout of available substrates for lean protein accretion (Patience et al., 2015). Finally, the observed fecal consistency scores are consistent with the pattern in ATTD of OM, GE, and CP among groups and agree with fecal scores reported by Kiarie et al. (2018) and Choi et al. (2023), indicating that pigs were not exposed to an overt health challenge that could compromise performance or nutrient digestibility.

SCFA are primarily produced by microbial fermentation of resistant carbohydrates and fiber in the colon. Acetate, propionate, and butyrate account for most SCFA production in the hindgut and are quickly absorbed in the colon. SCFAs, especially butyrate, are major fuels for the colonic mucosa and are trophic to both the small and large intestinal mucosa (Jacobi and Odle, 2012). In the current study, colon acetate concentration tended to be greatest in the BZA group and intermediate in the BC50 group compared to the NC and AGP groups. Although not significantly different, colon butyrate exhibited the same pattern as colon acetate. The SCFA concentrations in this study largely align with Kiarie et al. (2018) and Silveira et al. (2018) who reported that acetate and butyrate concentrations in cecal digesta tended to be greater in pigs weaned at 21 d age and supplemented with benzoic acid. The potential modulatory effects of BC50 and BZA on the gut microbiome could contribute to differences in hindgut fermentation capacity and SCFA production (Kluge et al., 2006; Torrallardona et al., 2007).

Weaning alone, in the absence of a direct intestinal pathogen challenge, is linked to inappropriate or overproduction of proinflammatory cytokines (Hu et al., 2013). In turn, proinflammatory cytokines are associated with alterations in the structure of the small intestine and reductions in digestive enzyme activity (Pie et al., 2004). In the current study, plasma TNF-α concentration was not different among groups, indicating that systemic inflammatory status did not contribute to observed differences in growth performance and nutrient digestibility. The abundance of HMGB1, a proinflammatory cytokine that promotes the recruitment of immune cells to sites of injury or infection (Tang et al., 2023), was not different among groups in the jejunum, ileum, and colon. While the abundance of TNF-α, IL-1β, and IL-8 in the jejunum was also not different among groups, cytokine abundance declined over time, implying less intestinal inflammation. This is consistent with the decline in HMGB1 abundance across the ileum and colon from period 1 to period 2 and the measured increases in jejunum villus height and crypt depth as pigs recover from weaning stress (Pluske et al., 2018). Ileum and colon TNF-α abundance was lower in the BZA and BC50 groups, suggesting that BC50 mimics the anti-inflammatory effects of free benzoic in the gut. Conversely, IL-1β abundance in the colon was greatest in the BZA group, intermediate in the NC and BC50 groups, and lowest in the AGP group. The abundance of IL-8, a chemokine that mediates neutrophil chemotaxis to sites of infection and tissue damage, exhibited a similar pattern as IL-1β in the colon and suggests that BC50 partly restores the negative impact of BZA on colon IL-1β and IL-8 status. Notably, colon TNF-α abundance was greater in the AGP group than the BZA group, whereas colon IL-1β and IL-8 were lower in the AGP group compared to the BZA group. This discrepant response in colon proinflammatory cytokines between the AGP and BZA groups is not immediately clear.

The proinflammatory cytokines TNF-α and IL-1β directly affect intestinal permeability by modulating the expression and activity of myosin light chain kinase, an enzyme that disrupts the structure of the tight junction complex (Kaminsky et al., 2021). Collectively, proinflammatory cytokine abundances did not correspond to differences in the expression of the tight junction proteins CLDN3 and CLDN4. One possibility for this discrepancy is that measuring tight junction protein abundance does not capture the distribution of claudin proteins between the cytosolic and membrane-associated fractions (Deluco et al., 2021). Only membrane-associated claudin proteins within the tight junction complex participate in the regulation of intestinal permeability. Moreover, this result contrasts with Chen et al. (2017) who demonstrated increased jejunal expression of occludin and zona occludens-1 transcripts in pigs weaned at 21 d age and supplemented with benzoic acid. Nonetheless, the difference in TNF-α in the ileum and TNF-α, IL-1β, and IL-8 in the colon between pigs that received either BC50 or BZA compared to pigs that received no additive or antibiotic growth promoter, however, suggests a disparity in the basal inflammatory tone of the distal gut. Future work directly characterizing the intestinal response of starter pigs supplemented with BZA and BC50 to pathogens that can affect the small intestine, such as enterotoxigenic *E. coli*, *Salmonella*, or *Lawsonia*, is warranted.

In conclusion, dietary supplementation of BC50 at a ratio of one to three equivalents of BZA minimally increases ADG and ADFI in starter pigs. Although the exact mechanism for this increase is unclear, the local immune status of the distal ileum and colon in response to free and BC50 may contribute to the observed differences. Further research is needed to determine the optimal dosage and delivery mechanisms of BZA and its protected preparations for improved growth performance and gut health in starter pigs. These findings have implications for improving animal health and production while reducing reliance on antibiotics and zinc oxide.

## Supplementary Material

txad111_suppl_Supplementary_Material

## References

[CIT0001] AOAC Int. 2006. Official methods of analysis of AOAC International. Gaithersburg, MD: AOAC International.

[CIT0002] AVMA. 2020. AVMA Guidelines for the Euthanasia of Animals: 2020 Edition. Schaumburg, IL: American Veterinary Medical Association.

[CIT0003] Bankhead, P., M. B. Loughrey, J. A. Fernandez, Y. Dombrowski, D. G. McArt, P. D. Dunne, S. McQuaid, R. T. Gray, L. J. Murray, H. G. Coleman, et al. 2017. QuPath: Open source software for digital pathology image analysis. Sci. Rep. 7:16878. doi:10.1038/s41598-017-17204-5 29203879 PMC5715110

[CIT0004] Casewell, M., C. Friis, E. Marco, P. McMullin, and I. Phillips. 2003. The European ban on growth-promoting antibiotics and emerging consequences for human and animal health. J. Antimicrob. Chemother. 52:159–161. doi:10.1093/jac/dkg313 12837737

[CIT0005] Chen, J. L., P. Zheng, C. Zhang, B. Yu, J. He, J. Yu, J. Q. Luo, X. B. Mao, Z. Q. Huang, and D. W. Chen. 2017. Benzoic acid beneficially affects growth performance of weaned pigs which was associated with changes in gut bacterial populations, morphology indices and growth factor gene expression. J. Anim. Physiol. Anim. Nutr. (Berl). 101:1137–1146. doi:10.1111/jpn.12627 27747941

[CIT0006] Chen, Y. M., E. T. Helm, N. Gabler, J. M. Hostetter, and E. R. Burrough. 2020. Alterations in intestinal innate mucosal immunity of weaned pigs during porcine epidemic diarrhea virus infection. Vet. Pathol. 57:642–652. doi:10.1177/0300985820932140 32880235

[CIT0007] Choi, H., Y. Chen, F. Longo, and S. W. Kim. 2023. Comparative effects of benzoic acid and sodium benzoate in diets for nursery pigs on growth performance and acidification of digesta and urine. J. Anim. Sci. 101:skad116. doi:10.1093/jas/skad116 37115097 PMC10184693

[CIT0008] Chopra, A., W. G. Willmore, and K. K. Biggar. 2019. Protein quantification and visualization via ultraviolet-dependent labeling with 2,2,2-trichloroethanol. Sci. Rep. 9:13923. doi:10.1038/s41598-019-50385-9 31558752 PMC6763483

[CIT0009] Correa, F., D. Luise, M. Castillo, S. Peris, A. Palomo-Yague, P. Bosi, and P. Trevisi. 2021. Effect of dietary supplementation with a blend of protected aromatic compounds, including benzoic acid, on growth performance and faecal microbial profile of weaned piglets as an alternative to Zinc Oxide. Livest. Sci. 246:104455. doi:10.1016/j.livsci.2021.104455

[CIT0010] Deluco, B., K. R. Fourie, O. M. Simko, and H. L. Wilson. 2021. Localization of claudin-3 and claudin-4 within the small intestine of newborn piglets. Physiol. Rep. 9:e14717. doi:10.14814/phy2.14717 33523589 PMC7849452

[CIT0011] Diao, H., Z. B. Gao, B. Yu, P. Zheng, J. He, J. Yu, Z. Q. Huang, D. W. Chen, and X. B. Mao. 2016. Effects of benzoic acid (VevoVitall (R)) on the performance and jejunal digestive physiology in young pigs. J. Anim. Sci. Biotechnol. 7:32. doi:10.1186/s40104-016-0091-y 27239300 PMC4884408

[CIT0012] Eriksen, E. O., E. Kudirkiene, A. E. Christensen, M. V. Agerlin, N. R. Weber, A. Nodtvedt, J. P. Nielsen, K. T. Hartmann, L. Skade, L. E. Larsen, et al. 2021. Post-weaning diarrhea in pigs weaned without medicinal zinc: risk factors, pathogen dynamics, and association to growth rate. Porcine Health Manag. 7:54. doi:10.1186/s40813-021-00232-z 34627400 PMC8501929

[CIT0013] Faccin, J. E. G., F. Laskoski, L. F. Hernig, R. Kummer, G. F. R. Lima, U. A. D. Orlando, M. A. D. Goncalves, A. P. G. Mellagi, R. R. Ulguim, and F. P. Bortolozzo. 2020a. Impact of increasing weaning age on pig performance and belly nosing prevalence in a commercial multisite production system. J. Anim. Sci. 98. doi:10.1093/jas/skaa031 PMC7183176 32034395

[CIT0014] Faccin, J. E. G., M. D. Tokach, M. W. Allerson, J. C. Woodworth, J. M. DeRouchey, S. S. Dritz, F. P. Bortolozzo, and R. D. Goodband. 2020b. Relationship between weaning age and antibiotic usage on pig growth performance and mortality. J. Anim. Sci. 98:skaa363. doi:10.1093/jas/skaa363 33188416 PMC7755175

[CIT0015] FDA. 2012. The judicious use of medically important antimicrobial drugs in food-producing animals. Rockville, MD: Food and Drug Administration.

[CIT0016] Friedman, M., P. R. Henika, and R. E. Mandrell. 2003. Antibacterial activities of phenolic benzaldehydes and benzoic acids against *Campylobacter jejuni*, *Escherichia coli*, *Listeria monocytogenes*, and *Salmonella enterica*. J. Food Prot. 66:1811–1821. doi:10.4315/0362-028x-66.10.1811 14572218

[CIT0017] Gilbert, M. S., N. Ijssennagger, A. K. Kies, and S. W. C. van Mil. 2018. Protein fermentation in the gut; implications for intestinal dysfunction in humans, pigs, and poultry. Am. J. Physiol. Gastrointest. Liver Physiol. 315:G159–G170. doi:10.1152/ajpgi.00319.2017 29597354

[CIT0018] Graber, T., H. Kluge, F. Hirche, J. Broz, and G. I. Stangl. 2012. Effects of dietary benzoic acid and sodium-benzoate on performance, nitrogen and mineral balance and hippuric acid excretion of piglets. Arch. Anim. Nutr. 66:227–236. doi:10.1080/1745039x.2012.676812 22724168

[CIT0019] Heo, J. M., F. O. Opapeju, J. R. Pluske, J. C. Kim, D. J. Hampson, and C. M. Nyachoti. 2013. Gastrointestinal health and function in weaned pigs: a review of feeding strategies to control post-weaning diarrhoea without using in-feed antimicrobial compounds. J. Anim. Physiol. Anim. Nutr. (Berl) 97:207–237. doi:10.1111/j.1439-0396.2012.01284.x 22416941

[CIT0020] Hu, C. H., K. Xiao, Z. S. Luan, and J. Song. 2013. Early weaning increases intestinal permeability, alters expression of cytokine and tight junction proteins, and activates mitogen-activated protein kinases in pigs. J. Anim. Sci. 91:1094–1101. doi:10.2527/jas.2012-5796 23230104

[CIT0021] Humphrey, D. C., J. R. Bergstrom, E. P. Calvo, S. L. Trabue, K. D. Scoggin, and L. L. Greiner. 2022. The effect of benzoic acid with or without a direct-fed microbial on the nutrient metabolism and gas emissions of growing pigs. J. Anim. Sci. 100:skac296. doi:10.1093/jas/skac296 36056812 PMC9667959

[CIT0022] Jacobi, S. K., and J. Odle. 2012. Nutritional factors influencing intestinal health of the neonate. Adv. Nutr. 3:687–696. doi:10.3945/an.112.002683 22983847 PMC3648750

[CIT0023] Jensen, J., M. M. Larsen, and J. Bak. 2016. National monitoring study in Denmark finds increased and critical levels of copper and zinc in arable soils fertilized with pig slurry. Environ. Pollut. 214:334–340. doi:10.1016/j.envpol.2016.03.034 27107257

[CIT0024] Kaminsky, L. W., R. Al-Sadi, and T. Y. Ma. 2021. IL-1beta and the intestinal epithelial tight junction barrier. Front. Immunol. 12:767456. doi:10.3389/fimmu.2021.767456 34759934 PMC8574155

[CIT0025] Kiarie, E., C. Voth, D. Wey, C. Zhu, P. Vingerhoeds, S. Borucki, and E. J. Squires. 2018. Comparative efficacy of antibiotic growth promoter and benzoic acid on growth performance, nutrient utilization, and indices of gut health in nursery pigs fed corn-soybean meal diet. Can. J. Anim. Sci. 98:868–874. doi:10.1139/cjas-2018-0056

[CIT0026] Kluge, H., J. Broz, and K. Eder. 2006. Effect of benzoic acid on growth performance, nutrient digestibility, nitrogen balance, gastrointestinal microflora and parameters of microbial metabolism in piglets. J. Anim. Physiol. Anim. Nutr. (Berl). 90:316–324. doi:10.1111/j.1439-0396.2005.00604.x 16867077

[CIT0027] Knarreborg, A., N. Miquel, T. Granli, and B. B. Jensen. 2002. Establishment and application of an in vitro methodology to study the effects of organic acids on coliform and lactic acid bacteria in the proximal part of the gastrointestinal tract of piglets. Anim Feed Sci Tech 99:131–140. doi:10.1016/s0377-8401(02)00069-x

[CIT0028] Kristensen, N. B., J. V. Norgaard, S. Wamberg, M. Engbaek, J. A. Fernandez, H. D. Zacho, and H. D. Poulsen. 2009. Absorption and metabolism of benzoic acid in growing pigs. J. Anim. Sci. 87:2815–2822. doi:10.2527/jas.2009-2003 19502503

[CIT0029] Lopez-Galvez, G., M. Lopez-Alonso, A. Pechova, B. Mayo, N. Dierick, and J. Gropp. 2021. Alternatives to antibiotics and trace elements (copper and zinc) to improve gut health and zootechnical parameters in piglets: a review. Anim. Feed Sci. Tech. 271:114727. doi:10.1016/j.anifeedsci.2020.114727

[CIT0030] Moeser, A. J., C. S. Pohl, and M. Rajput. 2017. Weaning stress and gastrointestinal barrier development: implications for lifelong gut health in pigs. Anim. Nutr. 3:313–321. doi:10.1016/j.aninu.2017.06.003 29767141 PMC5941262

[CIT0031] Murphy, D. P., J. V. O’Doherty, T. M. Boland, C. J. O’Shea, J. J. Callan, K. M. Pierce, and M. B. Lynch. 2011. The effect of benzoic acid concentration on nitrogen metabolism, manure ammonia and odour emissions in finishing pigs. Anim. Feed Sci. Tech. 163:194–199. doi:10.1016/j.anifeedsci.2010.10.009

[CIT0032] Myers, W. D., P. A. Ludden, V. Nayigihugu, and B. W. Hess. 2004. Technical note: a procedure for the preparation and quantitative analysis of samples for titanium dioxide. J. Anim. Sci. 82:179–183. doi:10.2527/2004.821179x 14753360

[CIT0033] Nguyen, D. H., K. Y. Lee, H. N. Tran, and I. H. Kim. 2019. Effect of a protected blend of organic acids and medium-chain fatty acids on growth performance, nutrient digestibility, blood profiles, meat quality, faecal microflora, and faecal gas emission in finishing pigs. Can. J. Anim. Sci. 99:448–455. doi:10.1139/cjas-2016-0174

[CIT0034] NRC. 2012. Nutrient Requirements of Swine. 11th rev ed. Washington, DC: National Academies Press.

[CIT0035] Nyachoti, C. M., F. O. Omogbenigun, M. Rademacher, and G. Blank. 2006. Performance responses and indicators of gastrointestinal health in early-weaned pigs fed low-protein amino acid-supplemented diets. J. Anim. Sci. 84:125–134. doi:10.2527/2006.841125x 16361499

[CIT0036] Patience, J. F., M. C. Rossoni-Serao, and N. A. Gutierrez. 2015. A review of feed efficiency in swine: biology and application. J. Anim. Sci. Biotechnol. 6:33. doi:10.1186/s40104-015-0031-2 26251721 PMC4527244

[CIT0037] PHAC. 2015. Federal action plan on antimicrobial resistance and use in Canada: building on the federal framework for action. Ottawa, ON, Canada: Public Health Agency of Canada (PHAC). 10.14745/ccdr.v41is4a05 PMC5868712 29769968

[CIT0038] Pie, S., J. P. Lalles, F. Blazy, J. Laffitte, B. Seve, and I. P. Oswald. 2004. Weaning is associated with an upregulation of expression of inflammatory cytokines in the intestine of piglets. J. Nutr. 134:641–647. doi:10.1093/jn/134.3.641 14988461

[CIT0039] Pluske, J. R., D. L. Turpin, and J. C. Kim. 2018. Gastrointestinal tract (gut) health in the young pig. Anim. Nutr. 4:187–196. doi:10.1016/j.aninu.2017.12.004 30140758 PMC6104527

[CIT0040] Powell, S., T. D. Bidner, R. L. Payne, and L. L. Southern. 2011. Growth performance of 20- to 50-kilogram pigs fed low-crude-protein diets supplemented with histidine, cystine, glycine, glutamic acid, or arginine. J. Anim. Sci. 89:3643–3650. doi:10.2527/jas.2010-3757 21642498

[CIT0041] Rhouma, M., J. M. Fairbrother, F. Beaudry, and A. Letellier. 2017. Post weaning diarrhea in pigs: risk factors and non-colistin-based control strategies. Acta Vet. Scand. 59:31. doi:10.1186/s13028-017-0299-7 28526080 PMC5437690

[CIT0042] Rudar, M., A. Gachman, and M. Boersma. 2023. Technical note: simultaneous determination of amino thiols in pig tissue by ultra-high performance liquid chromatography with fluorescence detection. J. Anim. Sci. 101:skad017. doi:10.1093/jas/skad017 36630697 PMC9940738

[CIT0043] Silveira, H., L. G. M. Amaral, C. A. P. Garbossa, L. M. Rodrigues, C. C. D. Silva, and V. S. Cantarelli. 2018. Benzoic acid in nursery diets increases the performance from weaning to finishing by reducing diarrhoea and improving the intestinal morphology of piglets inoculated with Escherichia coli K88(). J. Anim. Physiol. Anim. Nutr. (Berl). 102:1675–1685. doi:10.1111/jpn.12977 30094927

[CIT0044] Slifierz, M. J., R. M. Friendship, and J. S. Weese. 2015. Methicillin-resistant *Staphylococcus aureus* in commercial swine herds is associated with disinfectant and zinc usage. Appl. Environ. Microbiol. 81:2690–2695. doi:10.1128/AEM.00036-15 25662976 PMC4375337

[CIT0045] Stein, H. H., B. Seve, M. F. Fuller, P. J. Moughan, C. F. M. de Lange; Committee on Terminology to Report, and Digestibility. 2007. Invited review: amino acid bioavailability and digestibility in pig feed ingredients: terminology and application. J. Anim. Sci. 85:172–180. doi:10.2527/jas.2005-742 17179553

[CIT0046] Suiryanrayna, M. V., and J. V. Ramana. 2015. A review of the effects of dietary organic acids fed to swine. J. Anim. Sci. Biotechnol. 6:45. doi:10.1186/s40104-015-0042-z 26500769 PMC4618844

[CIT0047] Tang, D., R. Kang, H. J. Zeh, and M. T. Lotze. 2023. The multifunctional protein HMGB1: 50 years of discovery. Nat. Rev. Immunol. doi:10.1038/s41577-023-00894-6 37322174

[CIT0048] Tang, K. L., N. P. Caffrey, D. B. Nobrega, S. C. Cork, P. E. Ronksley, H. W. Barkema, A. J. Polachek, H. Ganshorn, N. Sharma, J. D. Kellner, et al. 2017. Restricting the use of antibiotics in food-producing animals and its associations with antibiotic resistance in food-producing animals and human beings: a systematic review and meta-analysis. Lancet. Planet. Health. 1:e316–e327. doi:10.1016/S2542-5196(17)30141-9 29387833 PMC5785333

[CIT0049] Torrallardona, D., I. Badiola, and J. Broz. 2007. Effects of benzoic acid on performance and ecology of gastrointestinal ­microbiota in weanling piglets. Livest. Sci. 108:210–213. doi:10.1016/j.livsci.2007.01.062

[CIT0050] Toyo’oka, T. 2009. Recent advances in separation and detection methods for thiol compounds in biological samples. J. Chromatogr. B Analyt. Technol. Biomed. Life Sci. 877:3318–3330. doi:10.1016/j.jchromb.2009.03.034 19357000

[CIT0051] Tsukahara, T., R. Inoue, M. Nakatani, K. Fukuta, E. Kishino, T. Ito, and K. Ushida. 2016. Influence of weaning age on the villous height and disaccharidase activities in the porcine small intestine. Anim. Sci. J. 87:67–75. doi:10.1111/asj.12399 26153481

[CIT0052] Upadhaya, S. D., K. Y. Lee, and I. H. Kim. 2016. Effect of protected organic acid blends on growth performance, nutrient digestibility and faecal micro flora in growing pigs. J. Appl. Anim. Res. 44:238–242. doi:10.1080/09712119.2015.1031775

[CIT0053] Upadhaya, S. D., K. Y. Lee, S. Serpunja, T. H. Song, and I. H. Kim. 2018. Growth performance, nutrient digestibility, fecal microbiota and fecal noxious gas emission in weaning pigs fed high and low density diet with and without protected organic acid blends. Anim. Feed. Sci. Tech. 239:1–8. doi:10.1016/j.anifeedsci.2017.12.013

[CIT0054] Vahjen, W., D. Pietruszynska, I. C. Starke, and J. Zentek. 2015. High dietary zinc supplementation increases the occurrence of tetracycline and sulfonamide resistance genes in the intestine of weaned pigs. Gut Pathog. 7:23. doi:10.1186/s13099-015-0071-3 26322131 PMC4551370

[CIT0055] Warnecke, T., and R. T. Gill. 2005. Organic acid toxicity, tolerance, and production in Escherichia coli biorefining applications. Microb. Cell Fact. 4:25. doi:10.1186/1475-2859-4-25 16122392 PMC1208944

