# Supplementary material for: Evaluation of protected benzoic acid on growth performance, nutrient digestibility, and gut health indices in starter pigs
Source: Transl Anim Sci. 2023 Sep 14;7(1):txad111. doi: 10.1093/tas/txad111 (PMC10576511; doi:10.1093/tas/txad111)

Supplemental Figure S1. Original unedited representative chemiluminescent immunoblots (upper panel) and fluorescent total protein blots (lower panel) of CLDN3 (A), CLDN4 (B), and HMGB1 (C) in jejunum mucosa. Target protein abundance was normalized against total protein transferred onto the membrane by fluorescent modification of tryptophan residues on protein with 2,2,2-trichloroethanol. Additional blots were performed to analyze all samples (not shown); target protein abundance across blots was normalized to a pooled sample analyzed on each blot. Abbreviations used: CLDN4, claudin 3; CLDN4, claudin 4; HMGB1, high mobility group box 1; P, pooled sample.

Supplemental Figure S2. Original unedited representative chemiluminescent immunoblots (upper panel) and fluorescent total protein blots (lower panel) of CLDN3 (A), CLDN4 (B), and HMGB1 (C) in ileum mucosa. Target protein abundance was normalized against total protein transferred onto the membrane by fluorescent modification of tryptophan residues on protein with 2,2,2-trichloroethanol. Additional blots were performed to analyze all samples (not shown); target protein abundance across blots was normalized to a pooled sample analyzed on each blot. Abbreviations used: CLDN4, claudin 3; CLDN4, claudin 4; HMGB1, high mobility group box 1; P, pooled sample.

Supplemental Figure S3. Original unedited representative chemiluminescent immunoblots (upper panel) and fluorescent total protein blots (lower panel) of CLDN3 (A), CLDN4 (B), and HMGB1 (C) in colon mucosa. Target protein abundance was normalized against total protein transferred onto the membrane by fluorescent modification of tryptophan residues on protein with 2,2,2-trichloroethanol. Additional blots were performed to analyze all samples (not shown); target protein abundance across blots was normalized to a pooled sample analyzed on each blot. Abbreviations used: CLDN4, claudin 3; CLDN4, claudin 4; HMGB1, high mobility group box 1; P, pooled sample.

Fig. S1 A

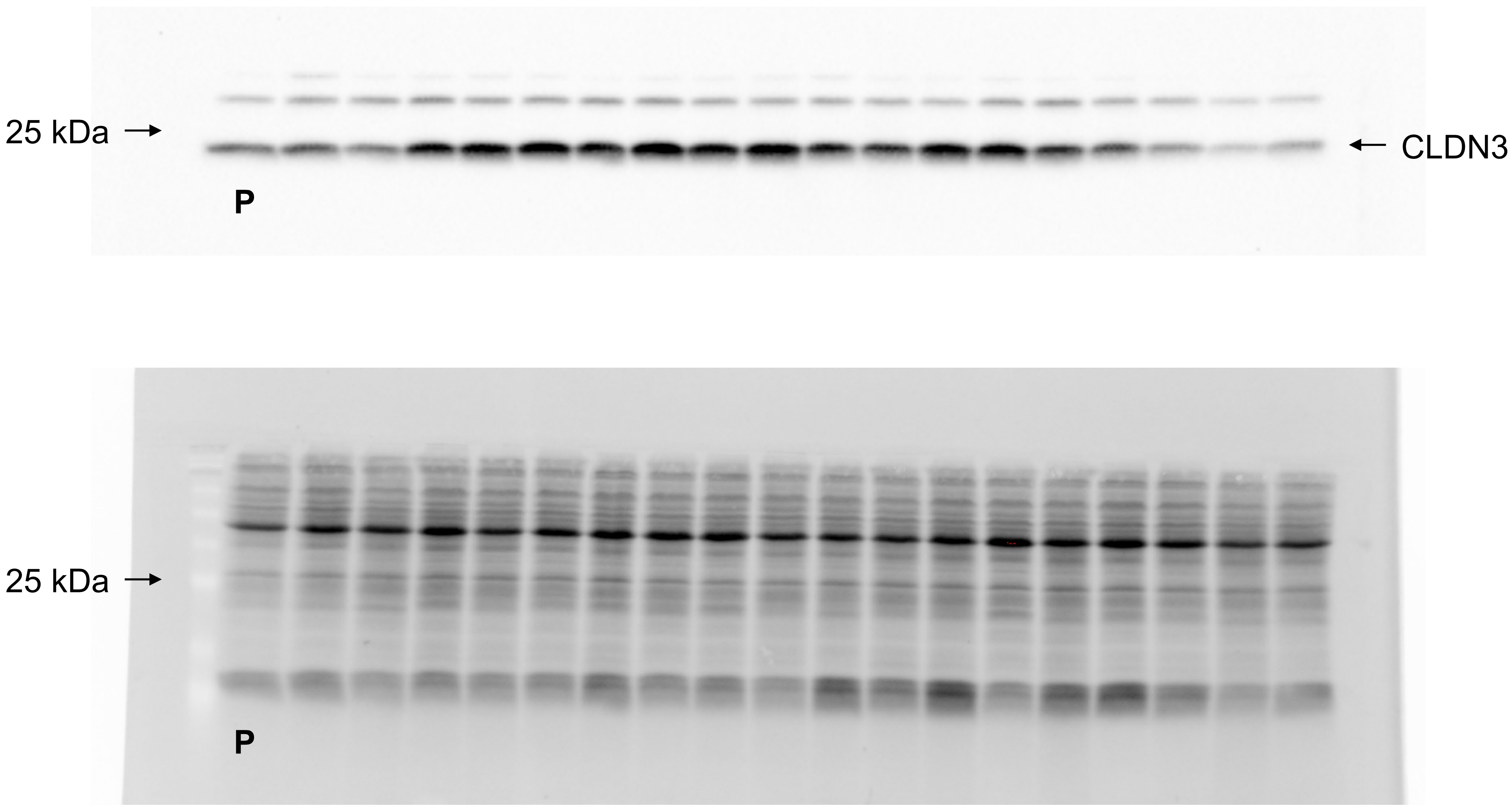

Fig. S1 B

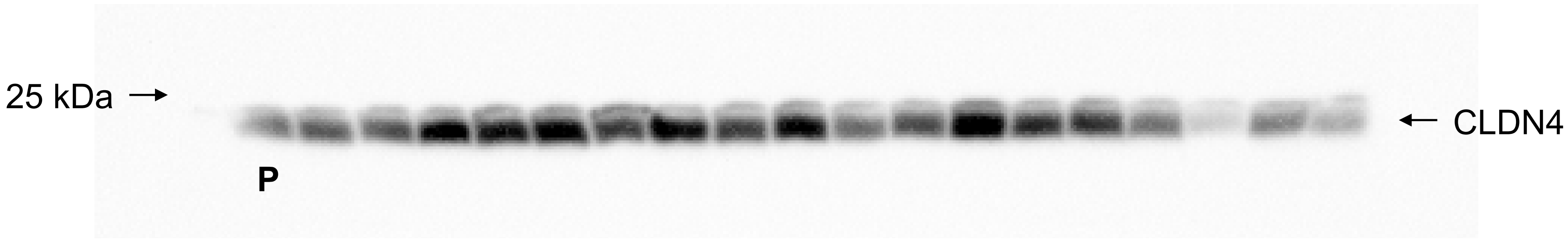

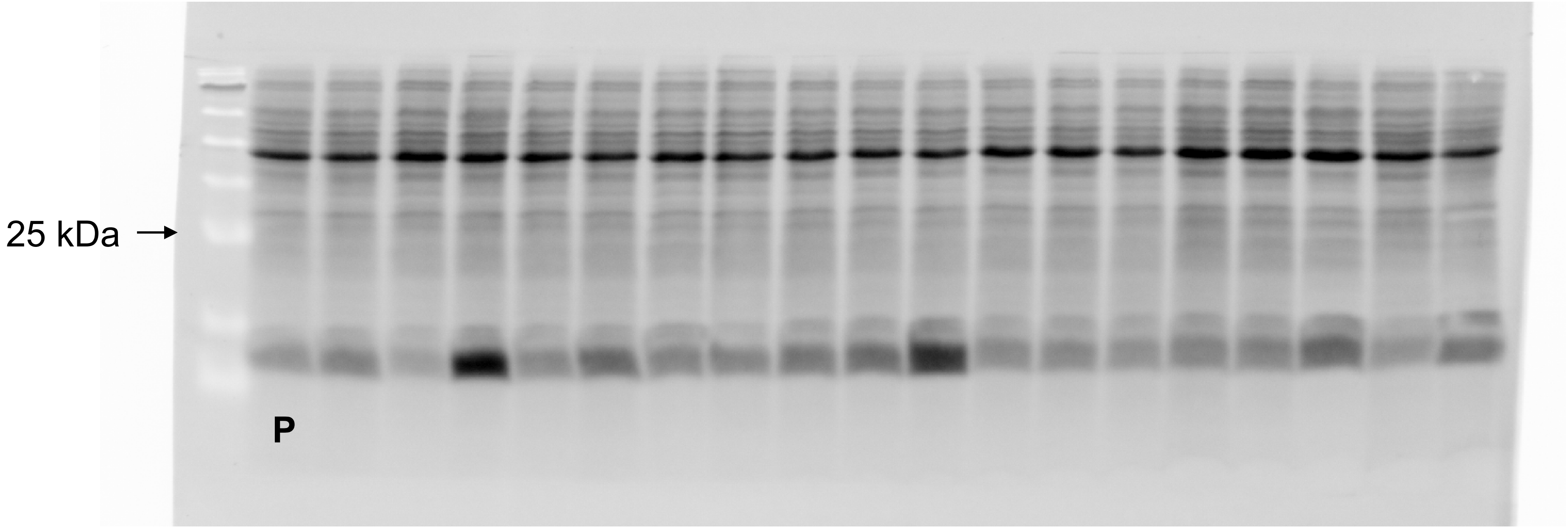

Fig. S1 C

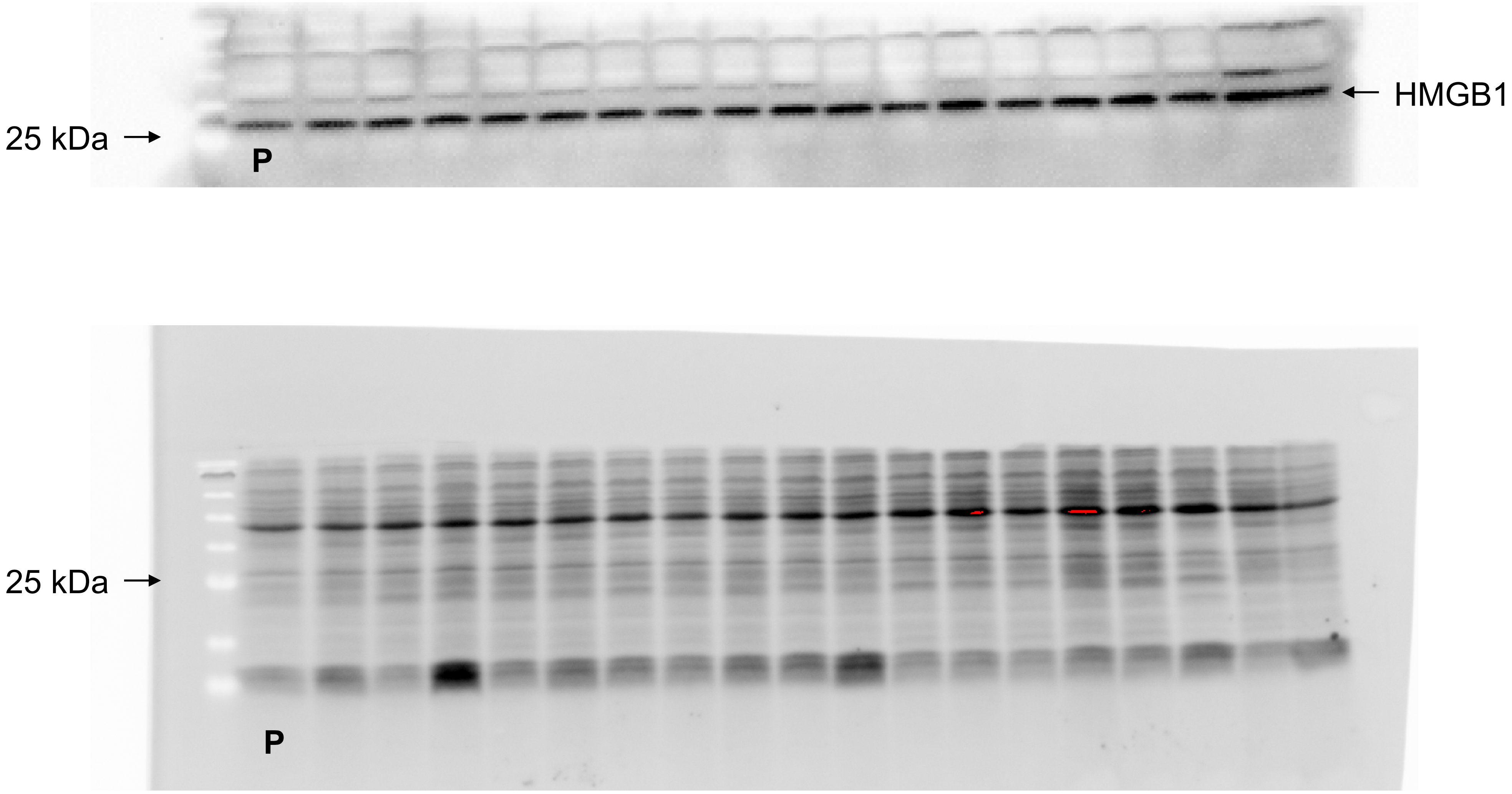

Fig. S2 A

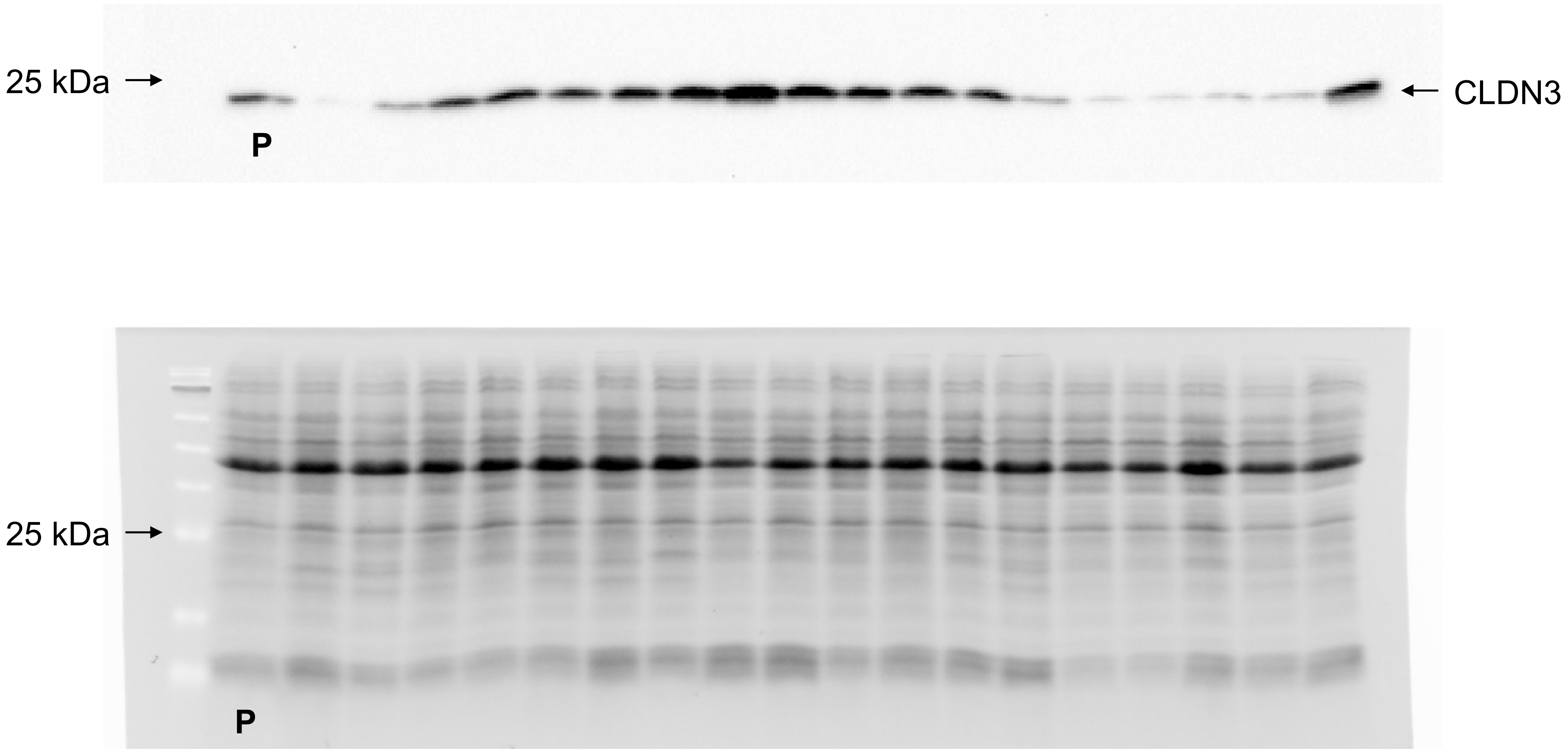

Fig. S2 B

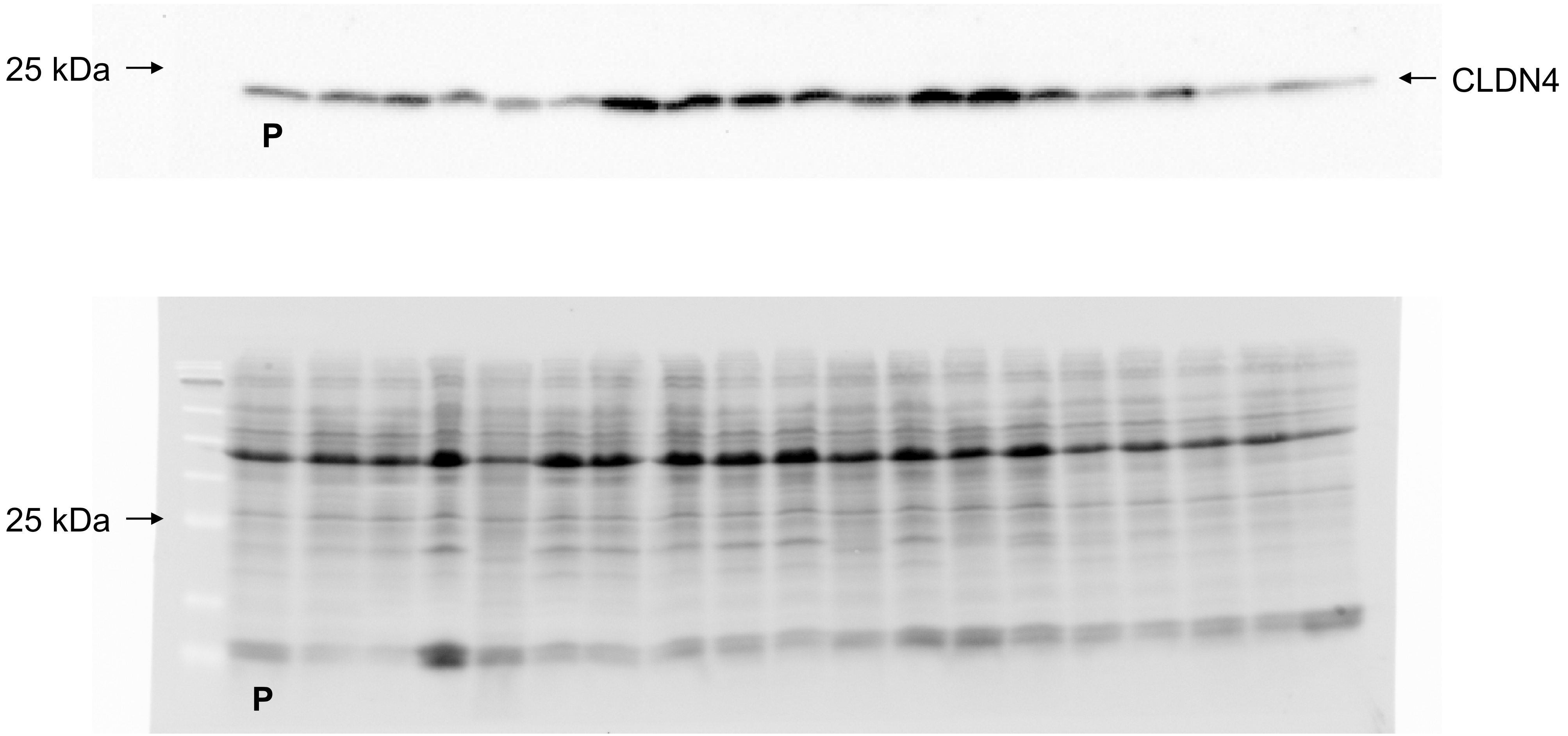

Fig. S2 C

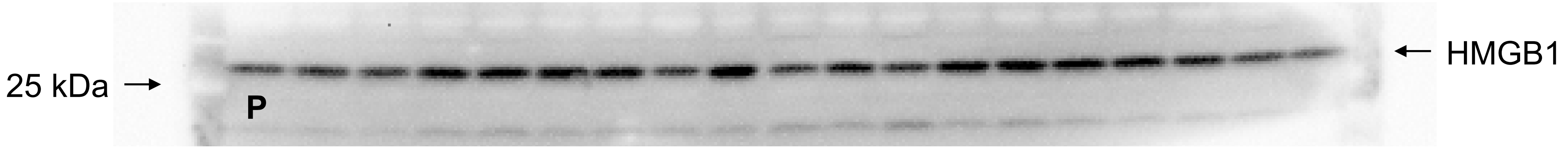

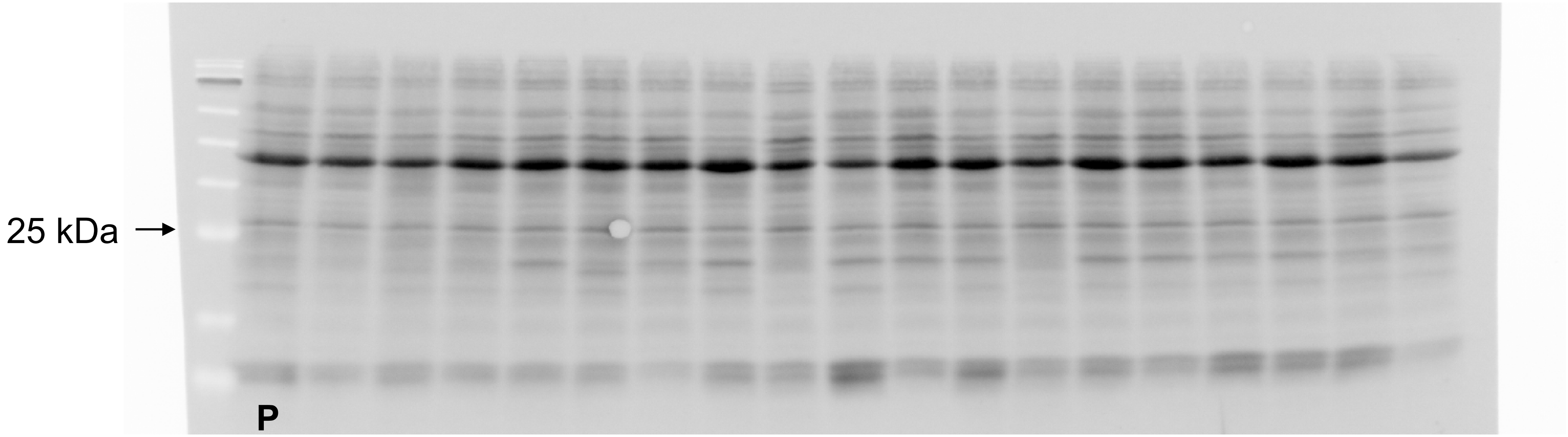

Fig. S3 A

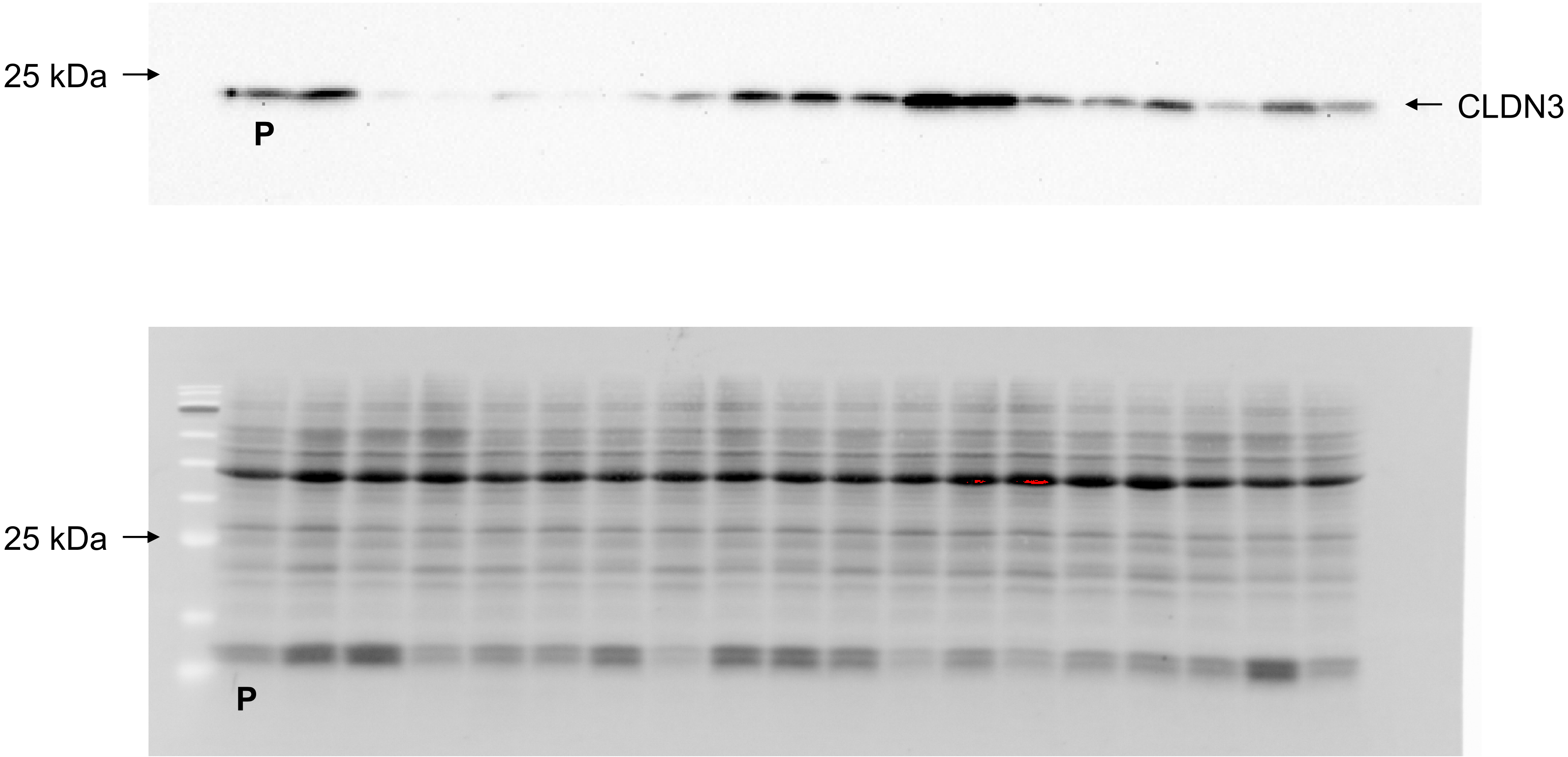

Fig. S3 B

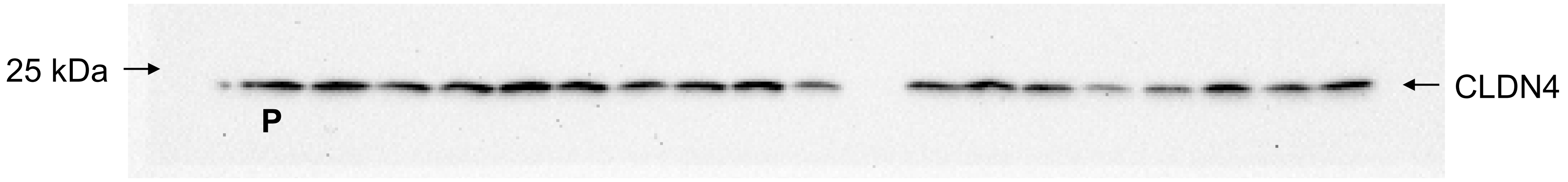

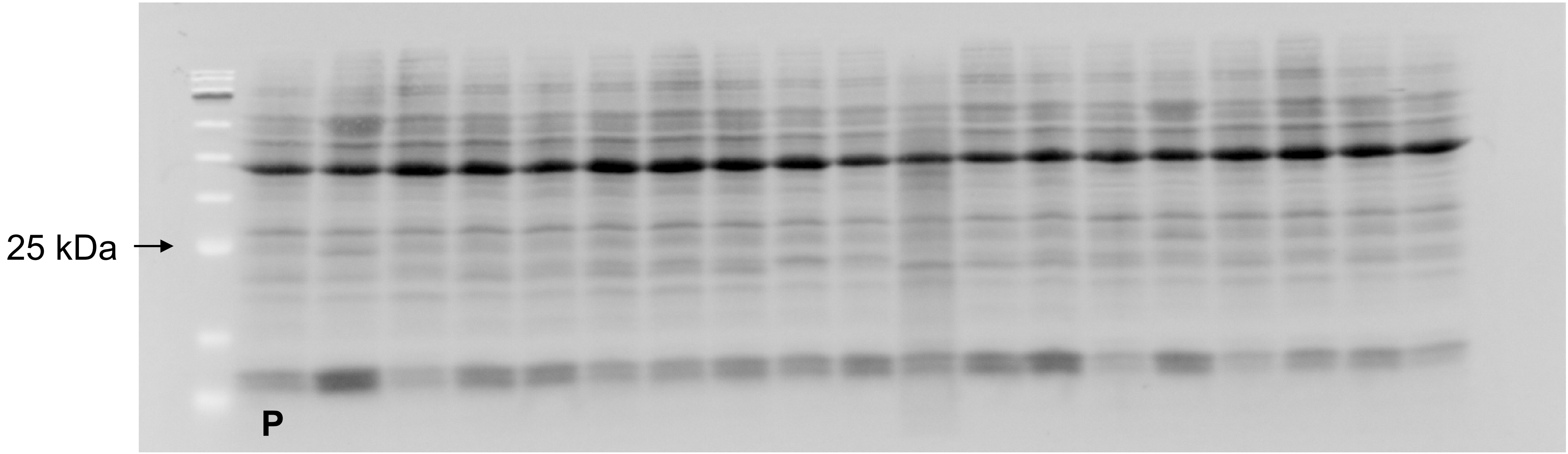

Fig. S3 C

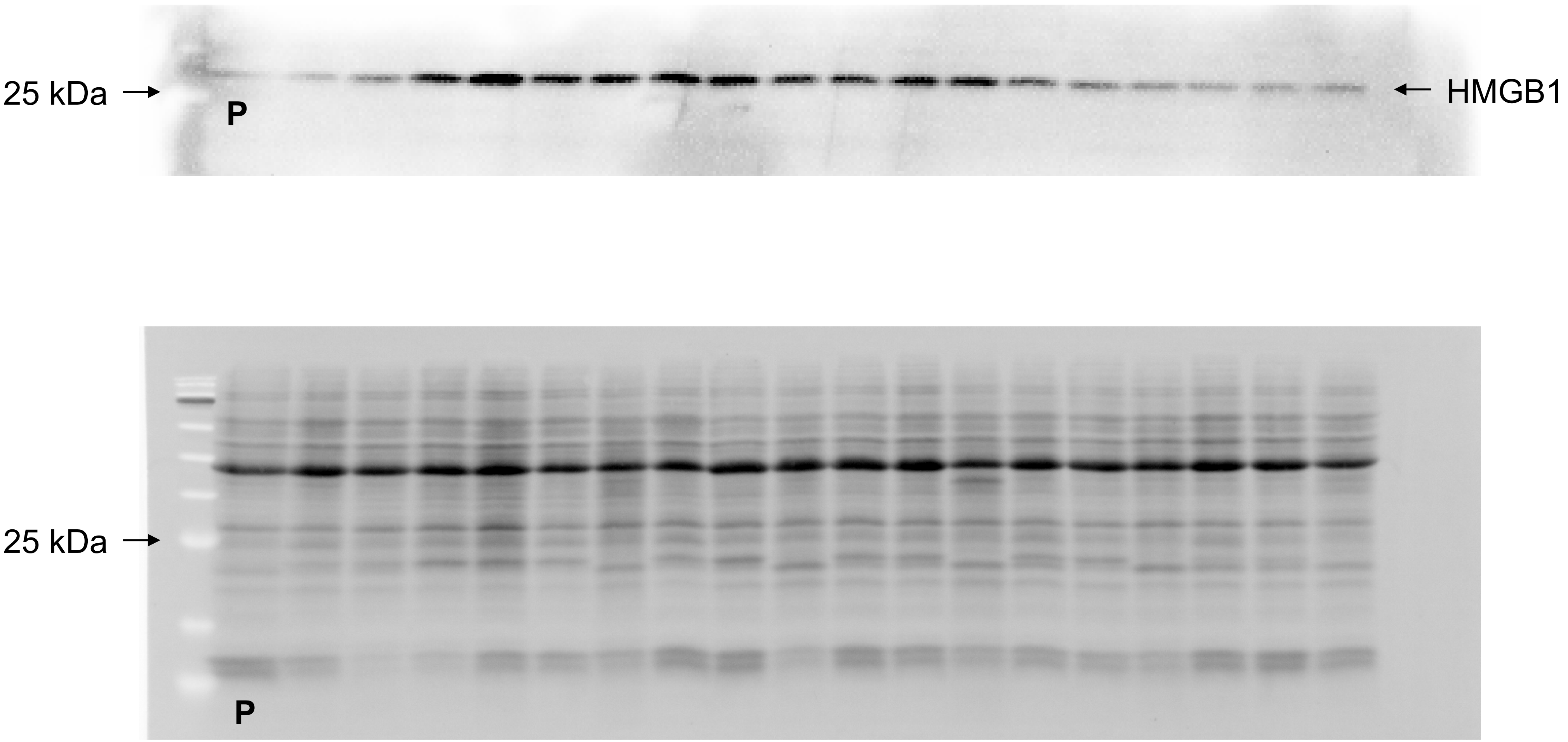

Supplement: txad111_suppl_Supplementary_Material [file txad111_suppl_supplementary_material.pdf]
